# rSeqDiff: Detecting Differential Isoform Expression from RNA-Seq Data Using Hierarchical Likelihood Ratio Test

**DOI:** 10.1371/journal.pone.0079448

**Published:** 2013-11-18

**Authors:** Yang Shi, Hui Jiang

**Affiliations:** 1 Department of Biostatistics, University of Michigan, Ann Arbor, Michigan, United States of America; 2 Center for Computational Medicine and Bioinformatics, University of Michigan, Ann Arbor, Michigan, United States of America; Queen’s University Belfast, United Kingdom

## Abstract

High-throughput sequencing of transcriptomes (RNA-Seq) has recently become a powerful tool for the study of gene expression. We present rSeqDiff, an efficient algorithm for the detection of differential expression and differential splicing of genes from RNA-Seq experiments across multiple conditions. Unlike existing approaches which detect differential expression of transcripts, our approach considers three cases for each gene: 1) no differential expression, 2) differential expression without differential splicing and 3) differential splicing. We specify statistical models characterizing each of these three cases and use hierarchical likelihood ratio test for model selection. Simulation studies show that our approach achieves good power for detecting differentially expressed or differentially spliced genes. Comparisons with competing methods on two real RNA-Seq datasets demonstrate that our approach provides accurate estimates of isoform abundances and biological meaningful rankings of differentially spliced genes. The proposed approach is implemented as an R package named rSeqDiff.

## Introduction

Alternative splicing is an important mechanism in post-transcriptional regulation of eukaryotes. Through alternative splicing, a single gene can produce multiple different transcript isoforms that usually lead to different protein isoforms with different structures and biological functions, which can greatly enrich the diversity of eukaryote transcriptomes [1]–[3]. Several studies also show that many human disease-causing mutations affect alternative splicing rather than directly affecting coding sequences and ill-regulated alternative splicing events have been implicated in a large number of human pathologies [4]–[6]. Due to its vital role in biological processes such as gene regulation, cell differentiation, development and disease pathophysiology, there is an urgent need for the development of new technologies and methodologies for the study of alternative splicing events and the quantification of the expression of alternative isoforms.

In recent years, high-throughput sequencing of transcriptomes (RNA-Seq) has rapidly evolved as a powerful tool for the study of alternative splicing in humans and model organisms [1]–[3], [7]. Many RNA-Seq experiments have been conducted to investigate the following two problems: (i) the discovery of novel transcripts and (ii) the estimation and detection of differentially expressed transcripts. Here we focus on the second problem. Several statistical approaches have been proposed in recent years towards this end. One type of approach is exon-based, which focuses on the detection of differential usage of exons [8]–[11]. The other type of approach is isoform-based, which focuses on the estimation of differential expression of isoforms across different biological conditions [12]–[16].

In this article, we present an isoform-based approach for the detection of differential isoform expression from multiple RNA-Seq samples. In particular, we extend the linear Poisson model in [17], [18] for the estimation of isoform abundances from single-end or paired-end RNA-Seq data. Unlike existing approaches which detect differential expression of transcripts, we consider three cases for each gene: 1) no differential expression, 2) differential expression without differential splicing and 3) differential splicing. We specify statistical models characterizing each of these three cases and use hierarchical likelihood ratio test for model selection. The remaining part of the paper is organized as follows: We first introduce the statistical model and method, and then use simulations to study the type-I error and statistical power of the proposed method, followed by the analyses of two real RNA-Seq datasets. For the first dataset (an ESRP1 dataset published in [11]), we compare our approach with two other methods (MATS [11] and Cuffdiff 2 [16]) using RT-PCR assays performed in [11]. For the second dataset (an ASD dataset published in [19]), we present a genome-widely analysis of differential splicing between Autism Spectrum Disorder (ASD) and normal brain samples.

## Methods

### Notations

We use similar notations as in [18] to present the statistical model, which are summarized in Table 1 and explained below in details.

**Table 1:** Summary of notations.

Symbol	Meaning
*K*	Total number of biological conditions in the study.
*I*	Total number of transcripts (isoforms) of a specific gene of interest.
*J_k_(J)*	Total number of read types in the *k*th condition (we write *J_k_* as *J* to avoid cluttering, but note this quantity depends on the condition *k*).
*A_k_*	The *I*×*J_k_* read sampling rate matrix for the *k*th condition.
*N_k_*	The *J_k_*×1 read count vector for the *k*th condition.
*θ*	The *K*×*I* isoform abundance matrix for all *K* conditions. The *k*th row corresponds to the isoform abundance vector for the *k*th condition.
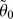	The *I*×1 joint isoform abundance vector for all *K* conditions (for model 0 only).
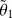	The *I*×1 basic isoform abundance vector (for model 1 only).
*τ*	The *K*×1 isoform ratio vector (for model 1 only).
*τ_k_*	The *k*th element of *τ* which is the ratio between the isoform abundance vector for the *k*th condition and the basic isoform abundance vector, i.e. *θ_k_* = *τ_k_* 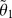 (for model 1 only).
*L_0_, L_1_, L_2_*	The likelihood functions for model 0, 1 and 2 (*l_0_*, *l_1_* and *l_2_* are the log-likelihood for each model), respectively.

### The linear Poisson model for multi-sample RNA-Seq data

We extend the linear Poisson model for one-sample RNA-Seq data in [17], [18] to multiple samples. Assume there are *K* conditions in the study, and in the *k*th condition there are *J_k_* distinct read types. A read type refers to a group of reads (single-end or paired-end) mapped to same position in a transcript [18]. We write *J_k_* as *J* to avoid cluttering but note this quantity depends on the condition *k*. For a gene *G* of interest with *I* annotated transcripts (isoforms), we define *θ* as the *K*×*I* isoform abundance matrix for all the *K* conditions, where the *k*th row vector of this matrix, 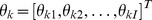 denotes the isoform abundance vector of *G* in the *k*th condition, and 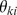 denotes the abundance of the *i*th isoform in the *k*th condition. Correspondingly, each condition has its own read sampling rate matrix
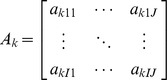
where 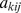 denotes the rate that read type *j* is sampled from isoform *i* in condition *k*. In our implementation we adopt the uniform sampling model in [18] for single-end reads which assumes all the possible read types from a transcript are generated with the same rate. For paired-end reads we adopt the insert length model in [18], which assumes the sampling rate of a particular paired-end read type depends on its insert size. The sampling rate matrix *A_k_* can be estimated based on all the mapped reads in condition *k* [18]. Each condition also has its own read count vector 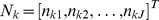, where *n_kj_* denotes the number of reads of type *j* mapped to any of the *I* isoforms in condition *k*. Given 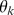 and *A_k_*, *N_k_* is assumed to follow the one-sample linear Poisson model [17], [18]. In particular, the probability mass function of *N_k_* is
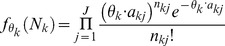
(1)where 
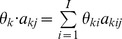
.

**Figure 1:**
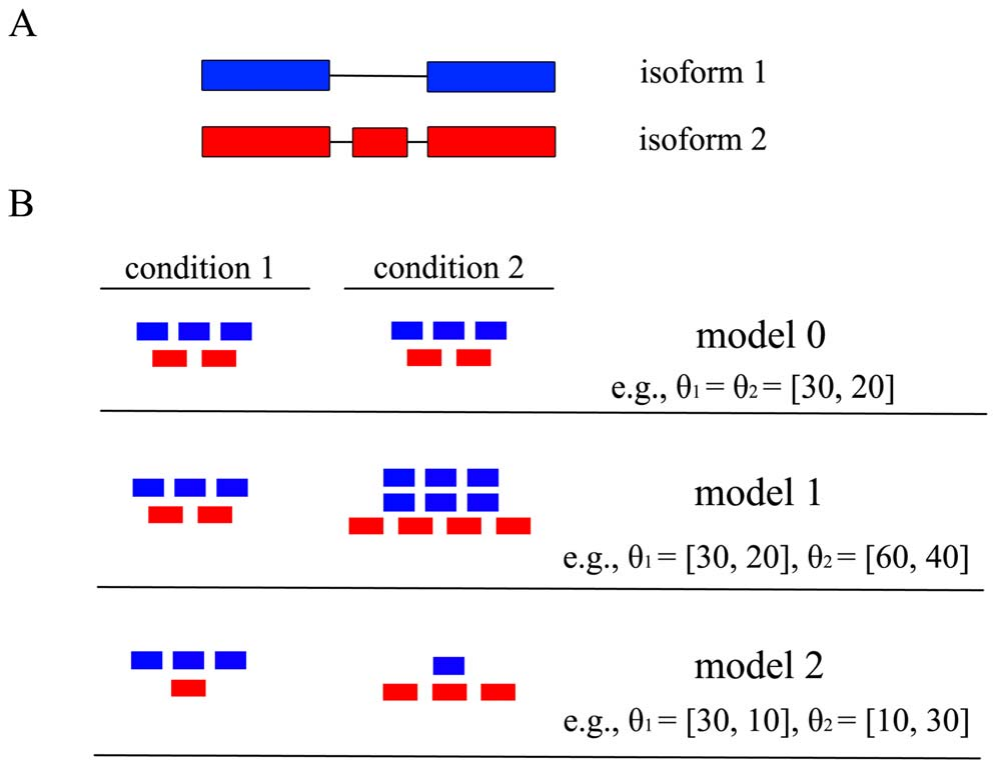
Illustration of the three models. (A) A hypothetical gene with three exons and two isoforms in blue and red, respectively. (B) Three models characterizing three biological situations of the gene expression patterns between two conditions. The numbers of red and blue bars represent the relative abundances of the corresponding isoforms in the two conditions.

Given *A_k_* and *N_k_* for *k* = 1, 2, …, *K*, our goal is to jointly estimate *θ* combining the data from all the samples. This will be complicated by the fact that the 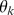
’s may not be independent of each other under different biological situations. Therefore, we need to re-parameterize *θ* according to the underlying biological situation of whether the gene and its isoforms show differential expression. In particular, we propose the following three nested models (Figure 1) corresponding to three possible underlying biological situations regarding the pattern of gene expression across multiple conditions.

*Model 0* [*no differential expression*] characterizes the situation where none of the gene’s isoforms show differential expression across the *K* conditions (Figure 1B, row 1, where the hypothetical gene structure is given in Figure 1A). Under this model, all *K* conditions have the same isoform expression levels so that all the rows of *θ* are the same and equal to a joint isoform abundance vector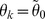, *k* = 1, 2, … *K*. Under the assumption that the reads of each condition are generated independently, the joint likelihood function of 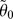 combining all *K* conditions is the product of the likelihood of each condition
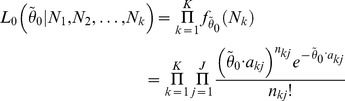
(2)


*Model 1* [*differential expression without differential splicing*] characterizes the situation where the gene shows differential expression, but not differential splicing of its isoforms across the *K* conditions (Figure 1B, row 2). Under this model, the relative abundances between the isoforms are the same across the *K* conditions and the rows of *θ* are therefore proportional to each other. Accordingly, we re-parameterize *θ* as the outer product of a *K*×1 vector *τ* and an *I*×1 vector 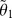, where 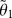 is the basic isoform abundance vector for all *K* conditions, and *τ* is the isoform ratio vector. To make the model identifiable, *τ* is subject to a linear constraint: 
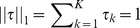
. For the example of model 1 in Figure 1B, 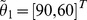 and 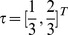. If 
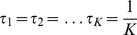
, model 1 degenerates to model 0. Similarly, the joint likelihood function of 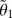 and *τ* combining all *K* conditions is
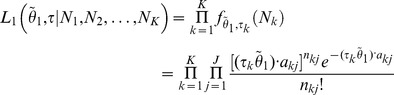
(3)


*Model 2* [*differential splicing*] characterizes the situation where the gene shows differential isoform usage across the *K* conditions (Figure 1B, row 3). Under this model, each condition has its own independent isoform abundance vector 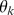. Therefore, the joint likelihood function is
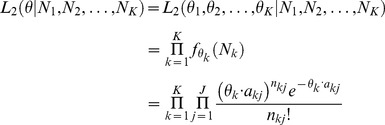
(4)


### Maximum likelihood estimation of the three models

The parameters of each of the three models can be estimated using maximum-likelihood estimation (MLE). As discussed in [18], one computational burden in solving the MLE is that *J* could be quite large, especially for paired-end RNA-Seq data. We adopt the two data reduction techniques introduced in [18]: (i) We take only read types with non-zero mapped reads and further group them to form larger read categories; (ii) For each condition *k*, we compute the total sampling rate for each isoform *i* 
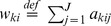
 (denote 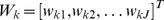
as the total sampling rate vector for all isoforms) without enumerating each particular sampling rate 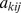. In practice, we work with the reduced form of the likelihood functions for the three models, and the details of these data reduction techniques are given in Text S1.

Similar to the log-likelihood function for one-sample linear Poisson model given in equation (1) (see also [17], [18]), all the log-likelihood functions for the above three models are concave. Therefore, the MLEs for all of the three models can be obtained by linear constraint convex optimization algorithms. In practice, we use an expectation-maximization (EM) algorithm to calculate the MLEs, and the details are given in Text S1.

### Model selection using hierarchical likelihood ratio test

Since model 0 is nested within model 1, which is again nested within model 2, we use the likelihood ratio test (LRT) for model selection. For large sample size, the LRT statistics for nested models asymptotically follow *χ^2^* distributions. The degrees of freedom (*DF*) of the three models are *DF*(model 0) = *I* (the free parameters are the *I*×1 joint isoform abundance vector 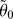), *DF*(model 1) = *I*+*K*-1 (the free parameters are the *I*×1 basic isoform abundance vector 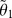 and the *K*×1 isoform abundance ratio vector *τ* subjects to one linear constraint 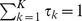) and *DF*(model 2) = *K*×*I* (the free parameters are the *K*×*I* isoform abundance matrix *θ*), respectively.

Given a pre-specified significance level α (e.g., 0.05), we perform model selection using the following hierarchical likelihood ratio test (hLRT) procedure (Table 2). The first round tests include two parallel tests which compare model 0 vs. model 1 and model 0 vs. model 2, each at significance level α/2. If neither of the two tests is significant, then model 0 is selected. If only one of the two tests is significant, model 1 or model 2 is selected accordingly. If both tests are significant, we perform the second round test which compares model 1 vs. model 2 at significance level α and selects model 2 if this test is significant or model 1 otherwise.

**Table 2:** Summary of hLRT for model selection.

	models beingcompared	LRTstatistics	testagainst
First round tests	model 0 vs. model 1	*−2(l0-l1)*	*χ^2^DF = K-*1, 1-α/2
	model 0 vs. model 2	*−2(l_0_-l_2_)*	*χ^2^DF = *(*K-*1)×*I*, 1-α/2
Second round test	model 1 vs. model 2	*−2(l_1_-l_2_)*	*χ^2^DF = *(*K-*1)×(*I*-1), 1-α

### Ranking of differentially spliced genes

When comparing between two biological conditions (e.g., normal vs. diseased), it is often useful to generate a ranking of genes being differentially spliced (i.e., model 2 genes). We rank model 2 genes as follows: Suppose 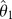 and 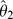 are the estimated isoform abundance vectors for the two conditions, we calculate the statistic:
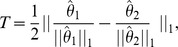
where ||·||_1_ denotes the vector *L_1_* norm ([20] uses a similar statistic without the constant 1/2, which is introduced here to have 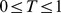). Large *T* values indicate high level of differential splicing. The *T* value is 0 for model 0 and model 1 genes. Alternatively, genes classified in model 1 or model 2 can also be ranked according to their p-values from the hLRT, if statistical significance is of major interest.

The proposed approach is implemented as an R package named rSeqDiff, which is available at http://www-personal.umich.edu/~jianghui/rseqdiff/. The analysis pipeline of using rSeqDiff is outlined in Figure S1 and Text S1.

## Results

### Simulation studies

We study the performance of our proposed hLRT approach by simulating read counts from genes with a wide range of abundances (from lowly expressed genes to highly expressed genes) and report the specificity and sensitivity of our approach for the detection of differential expression and differential splicing events. Detailed procedure and results of the simulation studies are given in Text S1, and here we briefly outline the methods that we applied in the simulations. We test differential expression and differential splicing of a hypothetical gene with a well-annotated known isoform structure (Figure S2) between two biological conditions with sequencing depths of total 50 million and 55 million reads, respectively. The gene structure and the sequencing depths are fixed in the simulations. For each of the three models, we vary the expression level (denoted as *G* in Text S1) of the gene within a broad range, and for each *G* we simulate the number of reads mapped to each of the two isoforms according to the three models (equations (2), (3) and (4)). For each *G*, we simulate 1000 replicated pairs of samples. We run the hLRT with significance level α = 0.05 using rSeqDiff on the 1000 simulated pairs of samples and report the proportions of the simulated pairs of samples for which our approach correctly selects the true underlying model (i.e., true classification rate). Table S1, S2 and S3 show the true classification rates under model 0, 1 and 2, respectively.

In summary, the simulation studies show that our proposed hLRT approach has well controlled type I error rate at α = 0.05 (Table S1) and good statistical power for detecting differential expression and differential splicing for genes with moderate to high abundance in both conditions (Table S2 and S3). When the gene is lowly expressed in one condition but moderately or highly expressed in the other condition, our proposed hLRT approach still has good power in selecting model 1, i.e., differential expression without differential splicing. The power in detecting differential expression or differential splicing is low when the gene has low expression levels in both conditions, which is well expected. In real data analysis, genes with very low expression levels in all the conditions are usually filtered out prior to the analysis. By default, rSeqDiff filters out genes with less than 5 reads in all the conditions.

### Applications of rSeqDiff to real RNA-Seq datasets

We demonstrate the practical usage of rSeqDiff and compare it with two other approaches by analyzing two real RNA-Seq datasets: the ESRP1 dataset and the ASD dataset.

#### Analysis of the ESRP1 dataset

Epithelial splicing Regulatory Protein 1 (ESRP1) is a master cell-type specific regulator of alternative splicing that controls a global epithelial-specific splicing network [11]. This dataset was published in [11], where Shen *et al* performed single-end RNA-Seq experiments on the MDA-MB-231 cell line with ectopic expression of the ESRP1 gene and an empty vector (EV) as control. The dataset contains 136 million reads for the ESRP1 sample and 120 million reads for the EV sample. Shen *et al* used this dataset to demonstrate their exon-based approach MATS for detect differential splicing, and performed RT-PCR assays to test for 164 exons skipping events. Since the biological significance of this dataset was further analyzed in a follow-up paper by Shen and collaborators [21], our analysis here is solely focused on the validation and comparisons of our proposed hLRT approach with other methods using the 164 RT-PCR tested alternative exons as gold standard.

**Figure 2:**
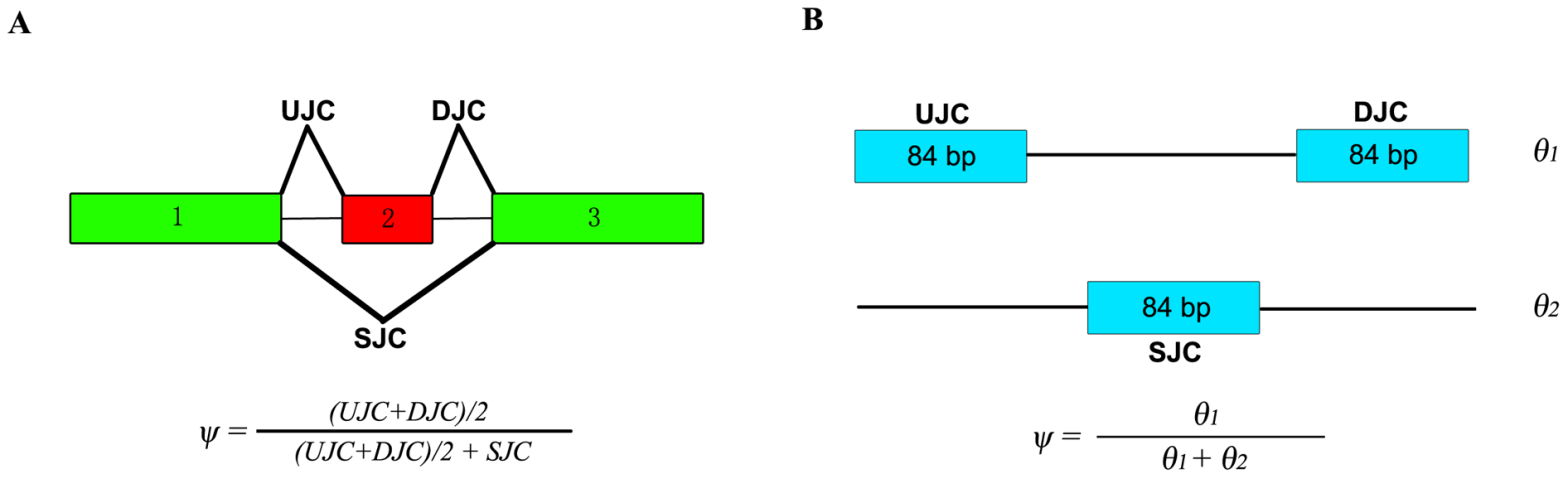
Models for estimating the exon inclusion level *ψ* using the junction reads. (A) The “exon-exon junction model” used by MATS [11]. Exon 1 and 3 are common exons shared by the two isoforms, and exon 2 is the skipped exon unique for the longer isoform. *ψ*: exon inclusion level; *UJC*: number of reads mapped to the upstream junction; *DJC*: number of reads mapped to the downstream junction; *SJC*: number of reads mapped to the skipping junction. (B) The “two-isoform model” transformed from (A). The abundances of the longer and shorter isoforms are *θ_1_* and *θ_2_*, respectively, which are estimated using the junction read counts (*UJC*, *DJC* and *SJC*).

**Figure 3:**
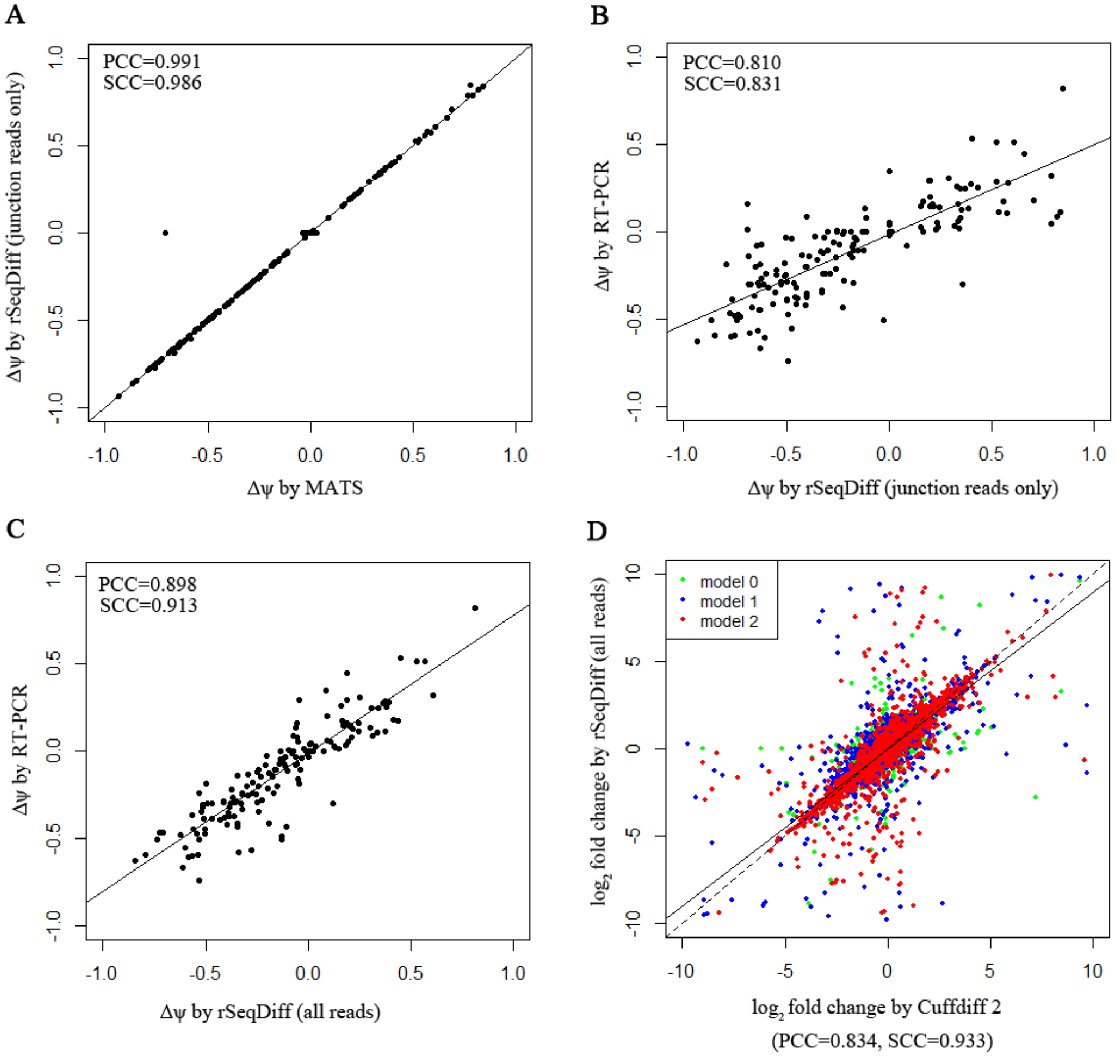
Comparisons of rSeqDiff, MATS, Cuffdiff 2 and RT-PCR assays. (A) Scatter plot of the *Δψ* values estimated by rSeqDiff (using junction reads only) and MATS. (B) Scatter plot of the *Δψ* values estimated by rSeqDiff (using junction reads only) and RT-PCR. (C) Scatter plot of the *Δψ* values estimated by rSeqDiff (using all reads) and RT-PCR. (D) Scatter plot of the log2 fold changes of isoform abundances between ESRP1 and EV estimated by rSeqDiff and Cuffdiff 2. Transcripts classified as model 0, model 1 and model 2 are shown in green, blue and red, respectively. The solid line is the regression line. The dashed line is the y = x line, which represents perfect agreement of the two methods. *Δψ*: difference of exon inclusion level between ESRP1 and EV; PCC: Pearson Correlation Coefficient; SCC: Spearman Correlation Coefficient.

MATS is an exon-based method and its results cannot be directly compared with our isoform-based approach. In the MATS model (Figure 2A, adapted from [11]), exon 2 is the alternatively spliced exon (skipped exon) unique for the longer isoform and exon 1 and 3 are common exons shared by both of the two isoforms. The exon inclusion level 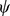 of the skipped exon was defined as the abundance ratio between the longer isoform and the sum of both the two isoforms, which was estimated as 
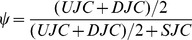
 by MATS (Figure 2A). The exon inclusion level difference between the two conditions (ESRP1 and EV) was calculated as 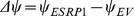. The genome coordinates, junctions read counts (*UJC*, *DJC* and *SJC*), 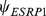, 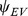 and 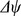 values from MATS and RT-PCR for the 164 exons are provided in [11]. We first apply rSeqDiff to these 164 exons using only the junction read counts from [11]. We transform the “exon-exon junction model” (Figure 2A) to a “two-isoform” model (Figure 2B), where the hypothetical “isoform 1” contains two “exons” each with length of 84 bp (the length of the exon-exon junction region in [18]) corresponding to the upstream junction (*UJC*) and downstream junction (*DJC*), respectively, and the hypothetical “isoform 2” contains a single “exon” with length of 84 bp corresponding to the skipping junction (*SJC*). Hence, the abundances of “isoform 1” (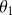) and “isoform 2” (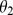) (Figure 2B) are equivalent to the abundances of the longer and shorter isoforms in exon-based method (Figure 2A), respectively. The exon inclusion level 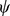 is then estimated as 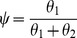. For the 164 RT-PCR tested exons, we first use rSeqDiff to estimate 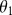 and 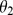 using the junction read counts (*UJC*, *DJC* and *SJC*) from [11], and then calculate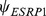, 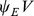 and 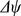 accordingly. Figure 3A shows the scatter plot of the 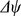 values estimated by rSeqDiff (using junction reads only) and MATS, and figure 3B shows the scatter plot of the 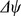 values estimated by rSeqDiff (using junction reads only) and RT-PCR (MATS and RT-PCR results are adapted from [11]). We can see that rSeqDiff gives very similar results as MATS when only junction reads are used, and overall both methods agree well with the RT-PCR assays (Figure 3B and Table 3).

**Table 3:** The correlation coefficients of the 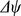 values between RT-PCR and rSeqDiff, MATS and Cuffdiff 2 for the 164 RT-PCR tested exons*.

	rSeqDiff (junction reads only)	rSeqDiff (all reads)	MATS*	Cuffdiff 2**
Pearson	0.810	**0.898**	0.799	0.838
Spearman	0.831	**0.913**	0.814	0.850

* The values from RT-PCR and MATS are directly adapted from [11].

** Three genes failed to be tested by Cuffdiff 2 (Reported as “FAIL”) are excluded.

We then apply rSeqDiff using its default settings (detailed method is given Text S1) where all the reads mapped to exons and exon-exon junctions are used (referred as rSeqDiff (all reads) below). We also run another isoform-based approach Cuffdiff 2 [16], [22] on the same dataset (details are given in Text S1). These two methods give the estimates of the abundances of all the isoforms. Based on the gene symbols and the genome coordinates of the 164 RT-PCR tested exons in [11], we identify genes containing these exons from the results of rSeqDiff (all reads) and Cuffdiff 2, and calculate the 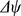 values for these exons based on the isoform abundances estimated by rSeqDiff (all reads) and Cuffdiff 2. Figure 3C shows the scatter plot of the 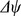 values estimated by rSeqDiff (all reads) and RT-PCR, and Table 3 shows the correlation coefficients of the 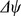 values between RT-PCR assays and the three methods, rSeqDiff, MATS and Cuffdiff 2, respectively. We can see that rSeqDiff (all reads) outperforms MATS and Cuffdiff 2 significantly.

**Figure 4:**
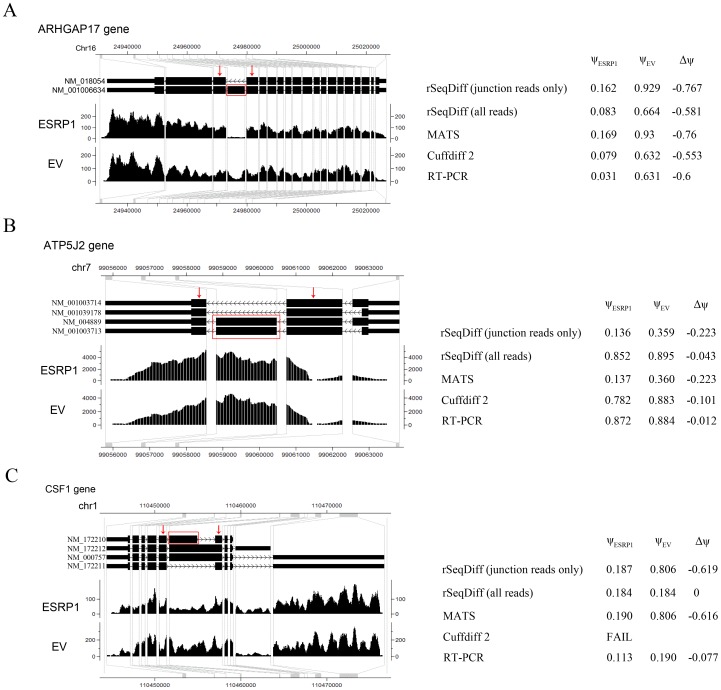
Examples comparing the estimates between rSeqDiff, MATS, Cuffdiff 2 and RT-PCR assays. (A) ARHGAP17 gene. (B) ATP5J2 gene. (C) CSF1 gene. The figures on the left show the gene structure and the coverage of reads mapped to the gene visualized in CisGenome Browser [45], where the horizontal tracks in the picture are (from top to bottom): genome coordinates, gene structures where introns are shrunken for better visualization and the coverage of reads mapped to the genes in ESRP1 and EV samples. The table to the right each figure shows the estimates from each method. 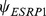 and 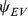: exon inclusion levels in ESRP1 and EV, respectively; *Δψ*: difference of exon inclusion levels between ESRP1 and EV (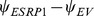).

One major advantage of isoform-based approaches like rSeqDiff and Cuffdiff 2 over exon-based approaches like MATS is that isoform-based approaches use all the reads mapped to exons and exon-exon junctions and incorporate the information from all the isoforms rather than using only the local exon structures as shown in figure 2A. The structure of the full length isoforms is important for inferring complex alternative splicing events. Three examples out of the 164 RT-PCR validated exons are given in Figure 4. In the first example (Figure 4A), the ARHGAP17 gene has only two isoforms differed by an alternative exon. The isoform structure of this gene is relative simple, and all the three algorithms provide similar estimates which are also validated by RT-PCR. In the second example (Figure 4B), the ATP5J2 gene has four isoforms differed by an alternative exon in the middle and an alternative 5′ splice site on the exon at the 5′ end. For this gene with a relative complex isoform structure, the two isoform-based methods, Cuffdiff 2 and rSeqDiff, give more accurate estimates than MATS, and rSeqDiff is slightly more accurate according to the RT-PCR result. In the third example (Figure 4C), the CSF1 gene has an even more complex isoform structure with four isoforms differed by an alternative exon in the middle and two mutually exclusive exons at the 3′ end. For such an isoform structure, some isoforms (NM_172212 and NM_000757) can only generate upstream junction reads (*UJC*) for the alternatively spliced middle exon but not downstream junction reads (*DJC*). As a result, the estimate of MATS is less accurate than that of rSeqDiff. rSeqDiff classifies this gene as model 1, which is consistent with the RT-PCR result. Cuffdiff 2 fails to test (it reports as “FAIL” [22]) this gene due to “an ill-conditioned covariance matrix or other numerical exception prevents testing”.

We also compare the estimates of all the gene between rSeqDiff (all reads) and Cuffdiff 2. Cuffdiff 2 fails to test (it reports as “LOWDATA”, “HIDATA” or “FAIL” [22]) several hundred genes with relative complex isoform structures. Figure 3D shows the scatter plot of the log2 fold changes of transcript abundances between ESRP1 and EV estimated by the two approaches (genes with low read counts or failed to be tested by Cuffdiff 2 are excluded). Overall the two approaches agree well with each other (Pearson Correlation Coefficient = 0.834, Spearman Correlation Coefficient = 0.933), and the degree of agreement is generally higher when the alternative spliced transcripts are more differentially expressed: the Pearson Correlation Coefficient (PCC) and Spearman Correlation Coefficient (SCC) of transcripts classified in each of the three models are PCC = 0.685, SCC = 0.802 (model 0), PCC = 0.827, SCC = 0.932 (model 1) and PCC = 0.862, SCC = 0.954 (model 2). We also run rSeqDiff with different fractions of reads from the dataset to check for possible saturation (Figure S3 and Table S4).

#### Analysis of the ASD dataset

Increasing evidence has indicated that alternative splicing plays an important role in brain development [23], [24] and the pathology of many neurological disorders [25], [26]. This dataset was published by Voineagu *et al* [19], where single-end RNA-Seq experiments were performed on three brain samples of Autism Spectrum Disorder (ASD) patients with down-regulated A2BP1 gene levels (a.k.a. FOX1, an important neuronal specific splicing factor that regulates alternative splicing in the brain) and three control brain samples with normal A2BP1 levels.

In [19], the authors separately pooled the reads for ASD and control to generate sufficient read coverage for the quantitative analysis of alternative splicing events (referred as “pooled dataset” below), and then used an exon-based method similar to MATS in their analysis and detected 212 significantly differentially spliced exons (belonging to 196 unique genes). As we have shown in the analysis of the ESRP1 dataset, the exon-based methods provide less accurate results for complex alternative splicing events and cannot infer the abundances of the isoforms, here we analyze this pooled dataset using rSeqDiff (detailed method is given in Text S1).

rSeqDiff classifies 4,507 genes (with 6,850 transcripts) as model 0, 12,374 genes (with 19,556 transcripts) as model 1, 1,769 genes (with 5,848 transcripts) as model 2 (Table S7), and 7,349 genes (with 8,884 transcripts) are filtered out because they have less than 5 mapped reads in both conditions (Figure S4). We also run Cuffdiff 2 [16], [22] on this dataset with its default settings. We find Cuffdiff 2 to be relatively conservative for detecting differential expression of spliced transcripts and it only identifies 43 transcripts as significant under default settings (FDR<0.05). Figure S5 shows the scatter plot of the log2 fold changes of transcript abundances between ASD and control estimated by the two approaches (genes with low read counts or failed to be tested by Cuffdiff 2 are excluded). Similar to the analysis of the ESRP1 dataset, the two methods generate concordant results overall (PCC = 0.825, SCC = 0.937). The correlation coefficients for transcripts classified in each of the three models are PCC = 0.539, SCC = 0.796 (model 0), PCC = 0.847, SCC = 0.940 (model 1) and PCC = 0.854, SCC = 0.953 (model 2), which also show the same pattern as we observed in the ESRP1 dataset. We also run rSeqDiff on each individual biological replicate and get consistent results as the analysis on the pooled dataset (Table S6).

The authors of [19] tested 7 differentially spliced exons with relevant neurological functions using semi-quantitative RT-PCR assays, and validated 6 of them. Table 4 shows the ranking of these genes by rSeqDiff and Cuffdiff 2 (The CDC42BPA gene was not validated in [19]). rSeqDiff is able to detect all the 6 confirmed genes as differentially spliced (model 2) and also gives a more meaningful ranking of these genes than Cuffdiff 2, which might be helpful for biologists to design follow-up experiments. We also compare the estimates of the exon inclusion levels of the six RT-PCR validated exons by rSeqDiff with the exon-based method in [19]. Five out of the six genes (except AGFG1) have concordant annotations for the skipped exons in the RefSeq annotation database are used in our analysis. Table S5 shows the comparisons between the two methods. Basically, rSeqDiff consistently recovers the results from the exon-based method in [19].

**Table 4:** Ranking of the RT-PCR validated genes with relevant neurological functions.

Genes	rSeqDiff	Cuffdiff 2
AGFG1	178	5841
RPN2	166	3884
EHBP1	281	8301
CDC42BPA*	Model 1	20470
GRIN1	338	6803
SORBS1	208	6313
NRCAM	325	FAIL**

* The RT-PCR result for this gene is not consistent with the exon-based method in [19], therefore this gene is not validated by RT-PCR. rSeqDiff classifies it in model 1.

** FAIL: the gene has “an ill-conditioned covariance matrix or other numerical exception that prevents testing” by Cuffdiff 2 [22].

We further analyze the biological significance of the differentially spliced genes detected by rSeqDiff. The enrichment of gene ontology (GO) terms and functional categories for the 1769 genes classified in model 2 are tested. 88 GO terms related to biological processes (Table S8), 48 GO terms related to cellular components (Table S9) and 30 functional categories (Table S10) are significantly enriched at FDR<0.01 level. rSeqDiff captures majority of the relevant enriched GO terms reported in [19], such as cell junction (*p* = 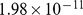), neuron projection (*p* = 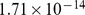), synapse (*p* = 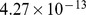) and clathrin-coated vesicle (*p* = 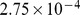) (Table S8 and S9). The result of enriched functional categories further confirms that alternative splicing is the top enriched functional category (*p* = 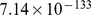) (Table S10). We further test the enrichment of genetic association disease classes (Table S11) and the tissue expression pattern of these genes (Table S12). Neuropsychiatric and neurological diseases are the two significantly enriched disease classes (*p* = 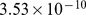 and 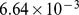 respectively, Table S11), and brain is the top enriched tissue (*p* = 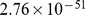, Table S12). We also search the relevance of the top 400 differentially spliced genes with autism and other related neurological diseases in the NIH Genetic Association Database [27] and the two autism spectrum disorder genetic database, AutDB [28] and SFARI Gene [29]. Among these genes, 173 are found to be associated with a variety of neurological and/or neuropsychiatric disorders from these databases, and 20 of them are associated with ASD (Table S7). Three of the 20 ASD-associated genes, NRCAM, EHBP1 and GRIN1, are validated by RT-PCR assays in [19]. All together, these results further demonstrate the biological significance of the findings of the differentially spliced genes.

**Figure 5:**
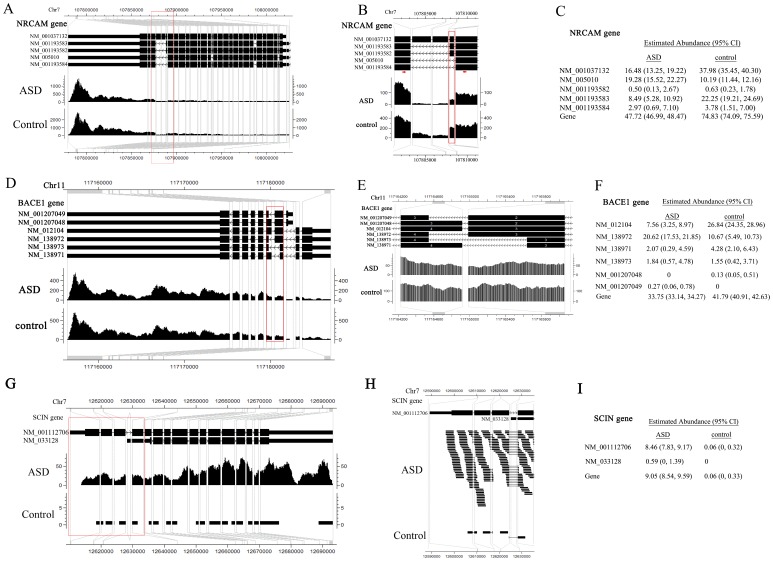
Examples demonstrating the estimates from rSeqDiff. (A)-(C) show NRCAM gene. (D)–(F) show BACE1 gene. (G)-(I) show SCIN gene. (A)(D)(G) show the gene structure and coverage of reads mapped to the gene. (B)(E)(H) show enlargement of the parts in the red boxes in (A)(D)(G), respectively, emphasizing the alternative spliced exons. In (B), the red box emphasizes the alternative exon that was validated by RT-PCR assay in [19], and the two red arrows represent the positions of the primers of RT-PCR [19]. (C)(F)(I) show estimated abundances for each gene and its isoforms by rSeqDiff. Values in the brackets are the 95% confidence intervals for the estimates.

Figure 5 shows three examples of genes with differential expression or differential splicing reported by rSeqDiff for the purpose of demonstrating rSeqDiff’s capability in dealing with very complex isoform structures. In the first example (Figure 5A–C), the NRCAM gene has five annotated alternative spliced isoforms (Figure 5A) and the estimation of their abundances between ASD and control is shown in Figure 5C. Figure 5B shows the differentially spliced exon that was validated by RT-PCR in [19]. This gene encodes a neuronal cell adhesion molecule which involves in neuron-neuron adhesion and promotes directional signaling during axonal cone growth [30] and has been reported to be associated with ASD by two genetic association studies [31], [32]. The second example is the BACE1 gene (Figure 5D–F) with six annotated alternative isoforms. This gene has a complex isoform structure, with an alternative 5′ splice site and an alternative 3′ splice site (the part in the red box of figure 5D, enlarged in figure 5E). The estimates of the abundances of the gene and its isoforms are shown in figure 5F. This gene encodes the β-site APP cleaving enzyme 1 (BACE1), which plays an important role in the pathology of Alzheimer’s disease [33]. Previous studies show that the isoforms of this gene have different enzymatic activities in the brain [34]–[36]. Although this gene has not been reported to be associated with ASD, several recent studies have showed that the expression levels of three BACE1 processed protein products, secreted amyloid precursor protein-α form (sAPP-α), secreted amyloid precursor protein-β form (sAPP-β) and amyloid-β peptide (Aβ), have substantial changes in severely autistic patients [37]–[40]. The third example is the SCIN gene (Figure 5G–I) with two alternative isoforms which differ by the mutually exclusive exons at the 5′ end (the part in the red box of figure 5G, enlarged in figure 5H). This gene is identified as model 1 by rSeqDiff, which has a significant higher expression level in autism than control. Also, there is no read mapped to the short exon unique to NM_033128 at its 5′ end (Figure 5H), therefore this isoform is estimated to have low abundances in both conditions. This gene encodes Scinderin (also known as Adseverin), a calcium-dependent actin filament severing protein that controls brain cortical actin network [41].

## Discussion

The two types of approaches for detecting differential transcription across multiple conditions, exon-based approaches and isoform-based approaches, each have their own strengths and weaknesses. Exon-based approaches do not rely on annotated full-length transcripts and provide relatively accurate inference for the differential splicing of a local exon from a gene with relative simple isoform structure [9], [11]. However, they cannot provide estimates of isoform abundances and provide less accurate inference for the differential splicing of genes with complex isoform structures. Isoform-based approaches can directly infer isoform abundances and are more accurate for estimating the differential splicing of multi-isoforms with complex splicing events. Since the final functional units are the protein isoforms translated from the alternatively spliced transcripts, isoform-based methods are more biologically informative for follow-up studies. However, isoform-based approaches may give inaccurate estimates if the annotation of full length transcripts is incorrect. We believe that isoform-based approaches will be increasingly used with the improvement of the transcript annotation databases.

One limitation of our approach is that it ignores the biological variations across biological replicates, which will be handled in our future work by extending our model. One way to handle biological variations is to use the negative binomial model as implemented in edgeR [42], DEseq [43], DSS [44] and Cuffdiff 2 [16], where an over-dispersion parameter is introduced and estimated using the empirical Bayes method that borrow information from all the genes. Another way is to use hierarchical Bayesian models, where choosing appropriate prior distributions and efficient parameter estimation (typically using Markov chain Monte Carlo (MCMC) algorithms) are challenging. It is also possible to extend our model to more complicated experimental designs such as crossed experiments by incorporating the covariates into the sampling rate matrix for each sample, since the hLRT is generally applicable to comparisons of complex models.

## Supporting Information

Figure S1 **The analysis pipeline by rSeqDiff.**
(DOC)

Figure S2 **A hypothetical gene used in simulations.** The length of the skipping exon (red) is 60 bp and the lengths of the two shared exons (green) are 1200 bp and 600 bp respectively. *θ*_11_ and *θ*_12_ denote the isoform abundances under condition 1 (50 million reads in total); *θ*_21_ and *θ*_22_ denote the isoform abundances under condition 2 (55 million reads in total).
(DOC)

Figure S3 **Performance of rSeqDiff with varying read numbers (corresponding to Table S4).** (A) Number of genes detected with reads greater than 5, model 0, model 1 and model 2 when using different proportions of reads. (B) Number of the genes among the 164 PCR tested genes detected when using different proportions of reads.
(DOC)

Figure S4 **Scatter plots for examining differential expression and differential splicing.** (A) Plot of the -log 10 based p values from the likelihood ratio test between model 1 and 0 v.s. the log2 fold changes of the estimated gene abundance, which can be used for visualizing differential expression of each gene. The red box highlights the SCIN gene that is shown as an example in the main text. (B) Plot of the -log 10 based p values from the likelihood ratio test between model 2 and 0 v.s the T values, which can be used for visualizing differential splicing of each gene. The red box highlights the BACE1 gene that is shown as an example in the main text.
(DOC)

Figure S5 **Comparison between rSeqDiff and Cuffdiff 2.** The log2 fold changes of isoform abundances between ASD and control samples estimated by rSeqDiff and Cuffdiff 2 are plotted. Transcripts classified as model 0, model 1 and model 2 are shown in green, blue and red, respectively. The solid line is the regression line. The dashed line is the y = x line, which represents perfect agreement of the two methods.
(DOC)

Table S1 **Summary of true classification rate under model 0 in simulations.**
(DOC)

Table S2 **Summary of true classification rate under model 1 in simulations.**
(DOC)

Table S3 **Summary of true classification rate under model 2 in simulations.**
(DOC)

Table S4 **Performance of rSeqDiff with varying read numbers.**
(DOC)

Table S5 **Comparison of the estimated differentially used exon inclusion levels for the five RT-PCR validated genes between rSeqDiff and the exon-based method in Voineagu** ***et al*** **.**
(DOC)

Table S6 **Comparison of differential spliced genes across biological replicates in the ASD dataset.**
(DOC)

Table S7 **rSeqDiff estimations for the 1769 genes classified as model 2, with the annotations of the relevant neurological diseases for the 173 genes among the top 400 genes.**
(XLS)

Table S8 **List of significantly enriched GO terms related to biological processes (BP) for the 1769 genes classified as model 2.**
(XLS)

Table S9 **List of significantly enriched GO terms related to cellular components (CC) for the 1769 genes classified as model 2.**
(XLS)

Table S10 **List of significantly enriched functional categories for the 1769 genes classified as model 2.**
(XLS)

Table S11 **List of the significantly enriched disease classes for the 1769 genes classified as model 2.**
(XLS)

Table S12 **List of the significantly enriched tissues for the 1769 genes classified as model 2.**
(XLS)

Text S1 **Supplementary methods and results.**
(DOC)

## References

[1] Wang ET, Sandberg R, Luo S, Khrebtukova I, Zhang L, et al (2008) Alternative isoform regulation in human tissue transcriptomes. Nature 456: 470–476. 18978772 10.1038/nature07509 PMC2593745

[2] Mortazavi A, Williams BA, McCue K, Schaeffer L, Wold B (2008) Mapping and quantifying mammalian transcriptomes by RNA-Seq. Nat Methods 5: 621–628. 18516045 10.1038/nmeth.1226 PMC13303166

[3] Pan Q, Shai O, Lee LJ, Frey BJ, Blencowe BJ (2008) Deep surveying of alternative splicing complexity in the human transcriptome by high-throughput sequencing. Nat Genet 40: 1413–1415. 18978789 10.1038/ng.259

[4] Lopez-Bigas N, Audit B, Ouzounis C, Parra G, Guigo R (2005) Are splicing mutations the most frequent cause of hereditary disease? FEBS Lett 579: 1900–1903. 15792793 10.1016/j.febslet.2005.02.047

[5] Kim E, Goren A, Ast G (2008) Insights into the connection between cancer and alternative splicing. Trends Genet 24: 7–10. 18054115 10.1016/j.tig.2007.10.001

[6] Luco RF, Allo M, Schor IE, Kornblihtt AR, Misteli T (2011) Epigenetics in alternative pre-mRNA splicing. Cell 144: 16–26. 21215366 10.1016/j.cell.2010.11.056 PMC3038581

[7] Garber M, Grabherr MG, Guttman M, Trapnell C (2011) Computational methods for transcriptome annotation and quantification using RNA-seq. Nat Methods 8: 469–477. 21623353 10.1038/nmeth.1613

[8] Katz Y, Wang ET, Airoldi EM, Burge CB (2010) Analysis and design of RNA sequencing experiments for identifying isoform regulation. Nat Methods 7: 1009–1015. 21057496 10.1038/nmeth.1528 PMC3037023

[9] Singh D, Orellana CF, Hu Y, Jones CD, Liu Y, et al (2011) FDM: a graph-based statistical method to detect differential transcription using RNA-seq data. Bioinformatics 27: 2633–2640. 21824971 10.1093/bioinformatics/btr458 PMC3179659

[10] Anders S, Reyes A, Huber W (2012) Detecting differential usage of exons from RNA-seq data. Genome Res 22: 2008–2017. 22722343 10.1101/gr.133744.111 PMC3460195

[11] Shen S, Park JW, Huang J, Dittmar KA, Lu ZX, et al (2012) MATS: a Bayesian framework for flexible detection of differential alternative splicing from RNA-Seq data. Nucleic Acids Res 40: e61. 22266656 10.1093/nar/gkr1291 PMC3333886

[12] Trapnell C, Williams BA, Pertea G, Mortazavi A, Kwan G, et al (2010) Transcript assembly and quantification by RNA-Seq reveals unannotated transcripts and isoform switching during cell differentiation. Nat Biotechnol 28: 511–515. 20436464 10.1038/nbt.1621 PMC3146043

[13] Vardhanabhuti S, Li M, Li H (2013) A Hierarchical Bayesian Model for Estimating and Inferring Differential Isoform Expression for Multi-Sample RNA-Seq Data. Stat Biosci 5: 119–137. 23737925 10.1007/s12561-011-9052-3 PMC3669631

[14] Trapnell C, Roberts A, Goff L, Pertea G, Kim D, et al (2012) Differential gene and transcript expression analysis of RNA-seq experiments with TopHat and Cufflinks. Nat Protoc 7: 562–578. 22383036 10.1038/nprot.2012.016 PMC3334321

[15] Glaus P, Honkela A, Rattray M (2012) Identifying differentially expressed transcripts from RNA-seq data with biological variation. Bioinformatics 28: 1721–1728. 22563066 10.1093/bioinformatics/bts260 PMC3381971

[16] Trapnell C, Hendrickson DG, Sauvageau M, Goff L, Rinn JL, et al (2013) Differential analysis of gene regulation at transcript resolution with RNA-seq. Nat Biotechnol 31: 46–53. 23222703 10.1038/nbt.2450 PMC3869392

[17] Jiang H, Wong WH (2009) Statistical inferences for isoform expression in RNA-Seq. Bioinformatics 25: 1026–1032. 19244387 10.1093/bioinformatics/btp113 PMC2666817

[18] Salzman J, Jiang H, Wong WH (2011) Statistical modeling of RNA-Seq data. Statistical Science 26: 62–83. 10.1214/10-STS343 PMC3846358 24307754

[19] Voineagu I, Wang X, Johnston P, Lowe JK, Tian Y, et al (2011) Transcriptomic analysis of autistic brain reveals convergent molecular pathology. Nature 474: 380–384. 21614001 10.1038/nature10110 PMC3607626

[20] Jiang H, Salzman J (2012) Statistical properties of an early stopping rule for resampling-based multiple testing. Biometrika 99: 973–980. 23843675 10.1093/biomet/ass051 PMC3629857

[21] Dittmar KA, Jiang P, Park JW, Amirikian K, Wan J, et al (2012) Genome-wide determination of a broad ESRP-regulated posttranscriptional network by high-throughput sequencing. Mol Cell Biol 32: 1468–1482. 22354987 10.1128/MCB.06536-11 PMC3318588

[22] Website of Cufflinks and Cuffdiff 2 package. Available: http://cufflinks.cbcb.umd.edu/manual.html. Accessed 2013 October 1.

[23] Li Q, Lee JA, Black DL (2007) Neuronal regulation of alternative pre-mRNA splicing. Nat Rev Neurosci 8: 819–831. 17895907 10.1038/nrn2237

[24] Norris AD, Calarco JA (2012) Emerging Roles of Alternative Pre-mRNA Splicing Regulation in Neuronal Development and Function. Front Neurosci 6: 122. 22936897 10.3389/fnins.2012.00122 PMC3424503

[25] Licatalosi DD, Darnell RB (2006) Splicing regulation in neurologic disease. Neuron 52: 93–101. 17015229 10.1016/j.neuron.2006.09.017

[26] Anthony K, Gallo JM (2010) Aberrant RNA processing events in neurological disorders. Brain Res 1338: 67–77. 20226177 10.1016/j.brainres.2010.03.008

[27] NIH Genetic Association Database. Available: http://geneticassociationdb.nih.gov/. Accessed 2013 October 1.

[28] Basu SN, Kollu R, Banerjee-Basu S (2009) AutDB: a gene reference resource for autism research. Nucleic Acids Res 37: D832–836. 19015121 10.1093/nar/gkn835 PMC2686502

[29] Simons Foundation Database for Autism Research. Available: https://gene.sfari.org/autdb/Welcome.do. Accessed 2013 October 1.

[30] Sakurai T (2012) The role of NrCAM in neural development and disorders–beyond a simple glue in the brain. Mol Cell Neurosci 49: 351–363. 22182708 10.1016/j.mcn.2011.12.002

[31] Sakurai T, Ramoz N, Reichert JG, Corwin TE, Kryzak L, et al (2006) Association analysis of the NrCAM gene in autism and in subsets of families with severe obsessive-compulsive or self-stimulatory behaviors. Psychiatr Genet 16: 251–257. 17106428 10.1097/01.ypg.0000242196.81891.c9

[32] Marui T, Funatogawa I, Koishi S, Yamamoto K, Matsumoto H, et al (2009) Association of the neuronal cell adhesion molecule (NRCAM) gene variants with autism. Int J Neuropsychopharmacol 12: 1–10. 18664314 10.1017/S1461145708009127

[33] Cole SL, Vassar R (2007) The Alzheimer’s disease beta-secretase enzyme, BACE1. Mol Neurodegener 2: 22. 18005427 10.1186/1750-1326-2-22 PMC2211305

[34] Mowrer KR, Wolfe MS (2008) Promotion of BACE1 mRNA alternative splicing reduces amyloid beta-peptide production. J Biol Chem 283: 18694–18701. 18468996 10.1074/jbc.M801322200

[35] Tanahashi H, Tabira T (2001) Three novel alternatively spliced isoforms of the human beta-site amyloid precursor protein cleaving enzyme (BACE) and their effect on amyloid beta-peptide production. Neurosci Lett 307: 9–12. 11516562 10.1016/s0304-3940(01)01912-7

[36] Zohar O, Cavallaro S, D’Agata V, Alkon DL (2003) Quantification and distribution of beta-secretase alternative splice variants in the rat and human brain. Brain Res Mol Brain Res 115: 63–68. 12824056 10.1016/s0169-328x(03)00182-7

[37] Ray B, Long JM, Sokol DK, Lahiri DK (2011) Increased secreted amyloid precursor protein-alpha (sAPPalpha) in severe autism: proposal of a specific, anabolic pathway and putative biomarker. PLoS One 6: e20405. 21731612 10.1371/journal.pone.0020405 PMC3120811

[38] Sokol DK, Chen D, Farlow MR, Dunn DW, Maloney B, et al (2006) High levels of Alzheimer beta-amyloid precursor protein (APP) in children with severely autistic behavior and aggression. J Child Neurol 21: 444–449. 16948926 10.1177/08830738060210062201

[39] Bailey AR, Giunta BN, Obregon D, Nikolic WV, Tian J, et al (2008) Peripheral biomarkers in Autism: secreted amyloid precursor protein-alpha as a probable key player in early diagnosis. Int J Clin Exp Med 1: 338–344. 19079679 PMC2596331

[40] Sokol DK, Maloney B, Long JM, Ray B, Lahiri DK (2011) Autism, Alzheimer disease, and fragile X: APP, FMRP, and mGluR5 are molecular links. Neurology 76: 1344–1352. 21482951 10.1212/WNL.0b013e3182166dc7 PMC3090060

[41] Trifaro JM, Rose SD, Marcu MG (2000) Scinderin, a Ca2+-dependent actin filament severing protein that controls cortical actin network dynamics during secretion. Neurochem Res 25: 133–144. 10685613 10.1023/a:1007503919265

[42] McCarthy DJ, Chen Y, Smyth GK (2012) Differential expression analysis of multifactor RNA-Seq experiments with respect to biological variation. Nucleic Acids Res 40: 4288–4297. 22287627 10.1093/nar/gks042 PMC3378882

[43] Anders S, Huber W (2010) Differential expression analysis for sequence count data. Genome Biol 11: R106. 20979621 10.1186/gb-2010-11-10-r106 PMC3218662

[44] Wu H, Wang C, Wu Z (2013) A new shrinkage estimator for dispersion improves differential expression detection in RNA-seq data. Biostatistics 14: 232–243. 23001152 10.1093/biostatistics/kxs033 PMC3590927

[45] Jiang H, Wang F, Dyer NP, Wong WH (2010) CisGenome Browser: a flexible tool for genomic data visualization. Bioinformatics 26: 1781–1782. 20513664 10.1093/bioinformatics/btq286 PMC2894522

