# Supplementary figures and images for: rSeqDiff: Detecting Differential Isoform Expression from RNA-Seq Data Using Hierarchical Likelihood Ratio Test
Source: PLoS One. 2013 Nov 18;8(11):e79448. doi: 10.1371/journal.pone.0079448 (PMC3832546; doi:10.1371/journal.pone.0079448)

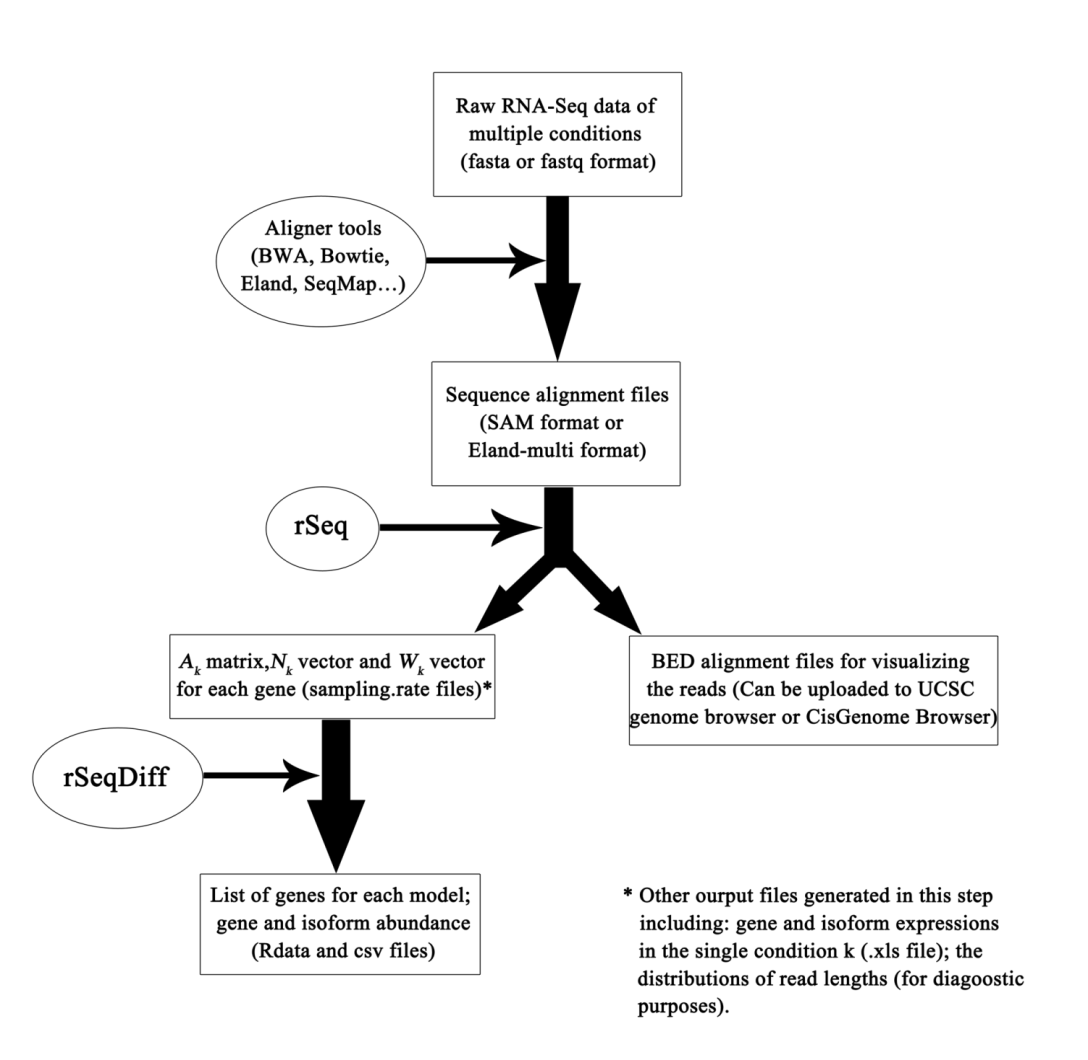


**Figure S1. The analysis pipeline by rSeqDiff.**

Supplement: Figure S1 — The analysis pipeline by rSeqDiff. (DOC) [file pone.0079448.s001.doc]
