# Supplementary material for: rSeqDiff: Detecting Differential Isoform Expression from RNA-Seq Data Using Hierarchical Likelihood Ratio Test
Source: PLoS One. 2013 Nov 18;8(11):e79448. doi: 10.1371/journal.pone.0079448 (PMC3832546; doi:10.1371/journal.pone.0079448)
Supplement: Figure S2 — A hypothetical gene used in simulations. The length of the skipping exon (red) is 60 bp and the lengths of the two shared exons (green) are 1200 bp and 600 bp respectively. θ 11 and θ 12 denote the isoform abundances under condition 1 (50 million reads in total); θ 21 and θ 22 denote the isoform abundances under condition 2 (55 million reads in total). (DOC) [file pone.0079448.s002.doc]

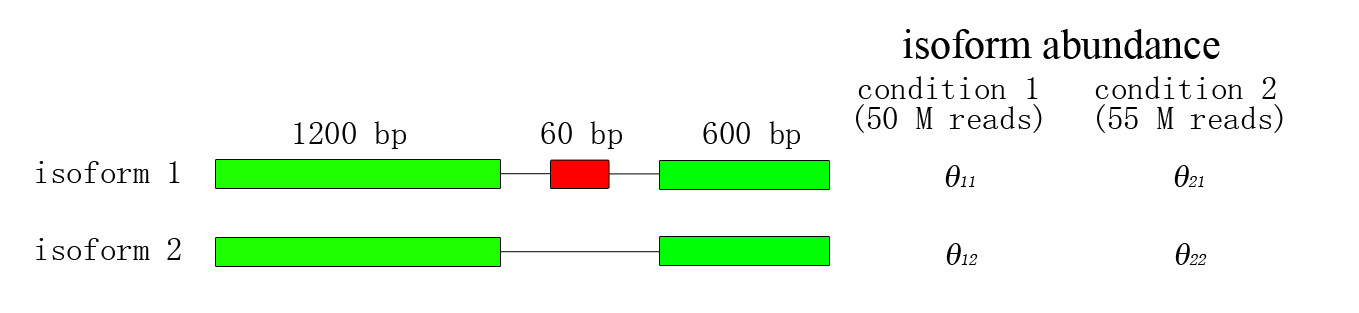


**Figure S2. A hypothetical gene used in simulations.** The length of the skipping exon (red) is 60 bp and the lengths of the two shared exons (green) are 1200 bp and 600 bp respectively. $\theta_{11}$and $\theta_{12}$ denote the isoform abundances under condition 1 (50 million reads in total); $\theta_{21}$ and $\theta_{22}$ denote the isoform abundances under condition 2 (55 million reads in total).
