# Supplementary material for: rSeqDiff: Detecting Differential Isoform Expression from RNA-Seq Data Using Hierarchical Likelihood Ratio Test
Source: PLoS One. 2013 Nov 18;8(11):e79448. doi: 10.1371/journal.pone.0079448 (PMC3832546; doi:10.1371/journal.pone.0079448)
Supplement: Figure S3 — Performance of rSeqDiff with varying read numbers (corresponding to Table S4). (A) Number of genes detected with reads greater than 5, model 0, model 1 and model 2 when using different proportions of reads. (B) Number of the genes among the 164 PCR tested genes detected when using different proportions of reads. (DOC) [file pone.0079448.s003.doc]

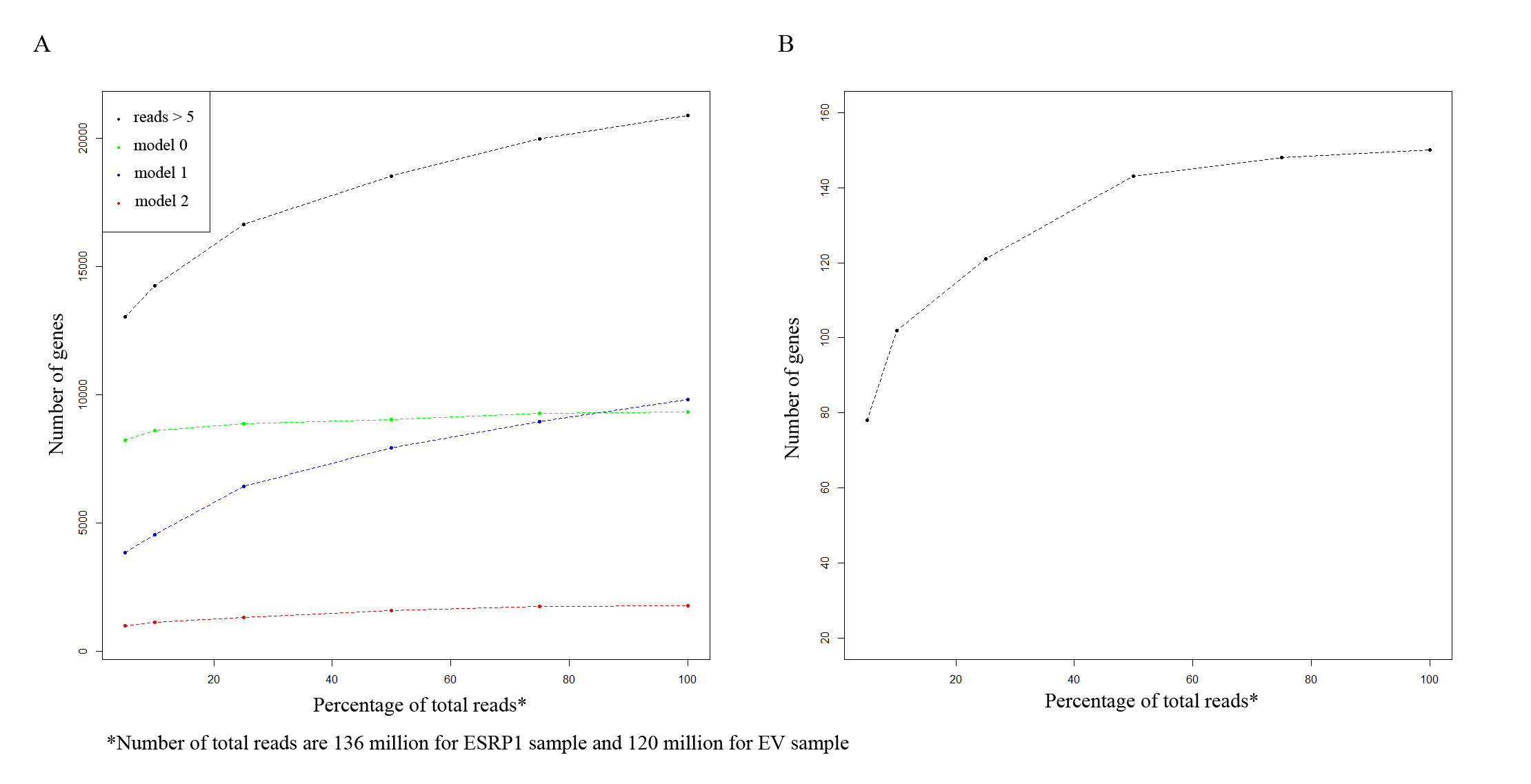


**Figure S3. Performance of rSeqDiff with varying read numbers (corresponding to Table S4).** (A) Number of genes detected with reads greater than 5, model 0, model 1 and model 2 when using different proportions of reads. (B) Number of the genes among the 164 PCR tested genes detectedwhen using different proportions of reads.
