# Supplementary material for: rSeqDiff: Detecting Differential Isoform Expression from RNA-Seq Data Using Hierarchical Likelihood Ratio Test
Source: PLoS One. 2013 Nov 18;8(11):e79448. doi: 10.1371/journal.pone.0079448 (PMC3832546; doi:10.1371/journal.pone.0079448)
Supplement: Figure S4 — Scatter plots for examining differential expression and differential splicing. (A) Plot of the -log 10 based p values from the likelihood ratio test between model 1 and 0 v.s. the log2 fold changes of the estimated gene abundance, which can be used for visualizing differential expression of each gene. The red box highlights the SCIN gene that is shown as an example in the main text. (B) Plot of the -log 10 based p values from the likelihood ratio test between model 2 and 0 v.s the T values, which can be used for visualizing differential splicing of each gene. The red box highlights the BACE1 gene that is shown as an example in the main text. (DOC) [file pone.0079448.s004.doc]

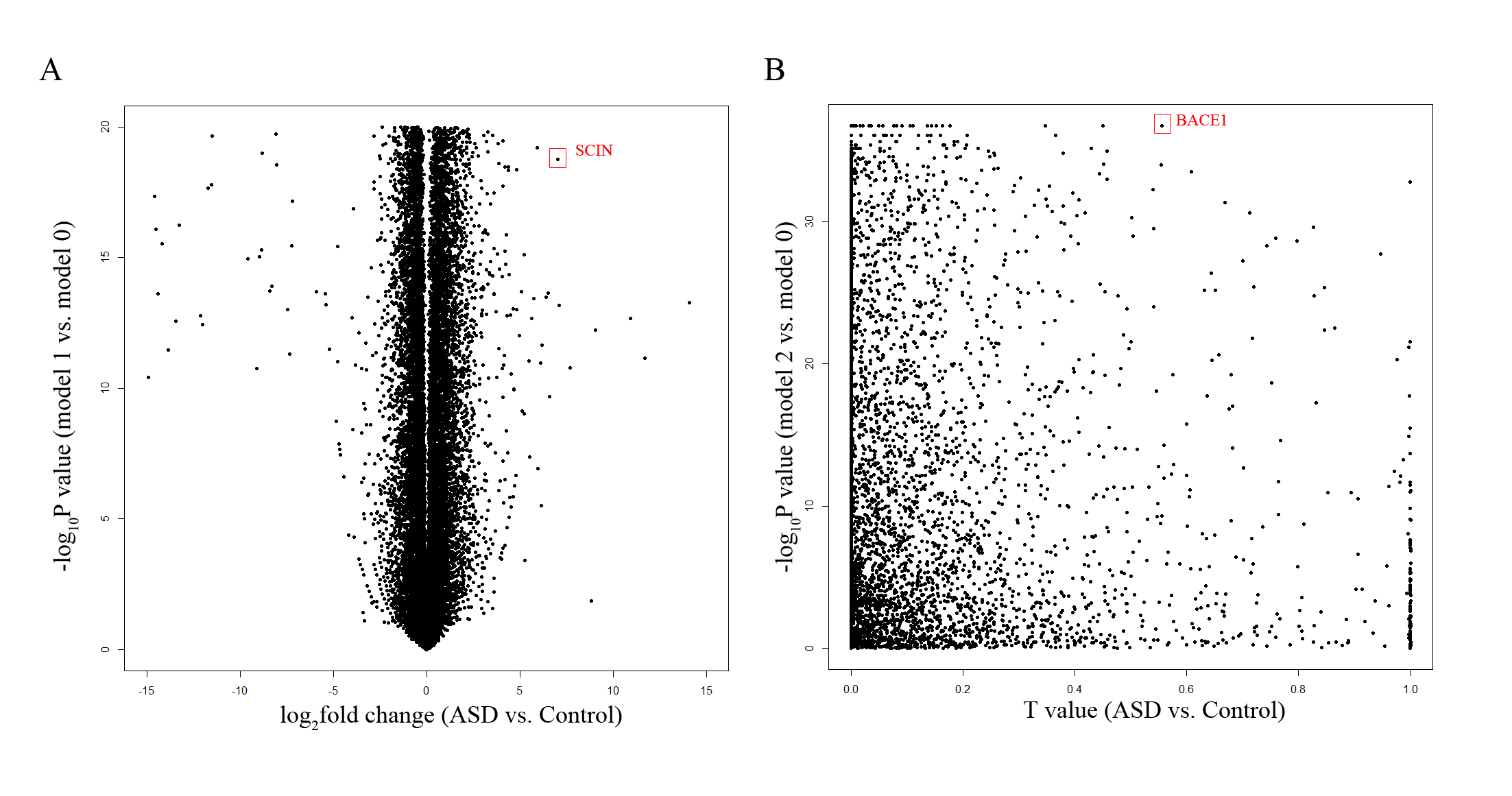


**Figure S4. Scatter plots for examining differential expression and differential splicing.** (A) Plot of the -log 10 based p values from the likelihood ratio test between model 1 and 0 v.s. the log2 fold changes of the estimated gene abundance, which can be used for visualizing differential expression of each gene. The red box highlights the SCIN gene that is shown as an example in the main text. (B) Plot of the -log 10 based p values from the likelihood ratio test between model 2 and 0 v.s the T values, which can be used for visualizing differential splicing of each gene. The red box highlights the BACE1 gene that is shown as an example in the main text.
