# Supplementary material for: rSeqDiff: Detecting Differential Isoform Expression from RNA-Seq Data Using Hierarchical Likelihood Ratio Test
Source: PLoS One. 2013 Nov 18;8(11):e79448. doi: 10.1371/journal.pone.0079448 (PMC3832546; doi:10.1371/journal.pone.0079448)
Supplement: Figure S5 — Comparison between rSeqDiff and Cuffdiff 2. The log2 fold changes of isoform abundances between ASD and control samples estimated by rSeqDiff and Cuffdiff 2 are plotted. Transcripts classified as model 0, model 1 and model 2 are shown in green, blue and red, respectively. The solid line is the regression line. The dashed line is the y = x line, which represents perfect agreement of the two methods. (DOC) [file pone.0079448.s005.doc]

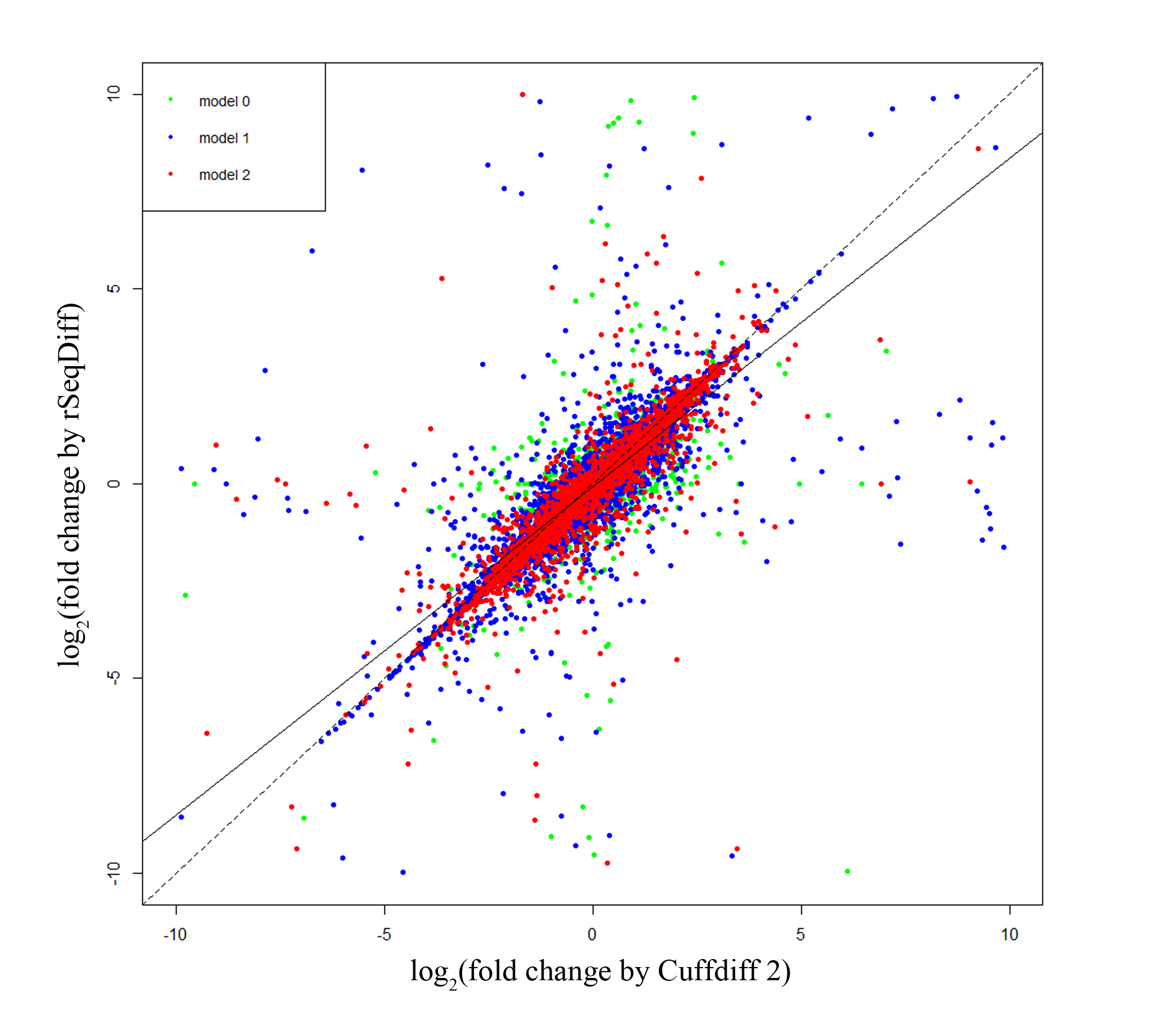


**Figure S5. Comparison between rSeqDiff and Cuffdiff 2.** The log2 fold changes of isoform abundances between ASD and control samples estimated by rSeqDiff and Cuffdiff 2 are plotted. Transcripts classified as model 0, model 1 and model 2 are shown in green, blue and red, respectively. The solid line is the regression line. The dashed line is the y=x line, which represents perfect agreement of the two methods.
