# Supplementary material for: rSeqDiff: Detecting Differential Isoform Expression from RNA-Seq Data Using Hierarchical Likelihood Ratio Test
Source: PLoS One. 2013 Nov 18;8(11):e79448. doi: 10.1371/journal.pone.0079448 (PMC3832546; doi:10.1371/journal.pone.0079448)
Supplement: Table S1 — Summary of true classification rate under model 0 in simulations. (DOC) [file pone.0079448.s006.doc]

**Table S1. Summary of true classification rate under model 0 in simulations.**

| *ψ*$G$ | 0.01 | 0.02 | 0.05 | 0.1 | 0.2 | 0.3 | 0.4 | 0.45 | 0.49 | 0.5 |
| --- | --- | --- | --- | --- | --- | --- | --- | --- | --- | --- |
| 0.1 | 0.968 | 0.966 | 0.97 | 0.974 | 0.971 | 0.966 | 0.973 | 0.964 | 0.974 | 0.984 |
| 1 | 0.97 | 0.978 | 0.972 | 0.974 | 0.966 | 0.954 | 0.954 | 0.944 | 0.965 | 0.952 |
| 10 | 0.97 | 0.972 | 0.954 | 0.944 | 0.973 | 0.954 | 0.972 | 0.958 | 0.951 | 0.963 |
| 100 | 0.962 | 0.958 | 0.958 | 0.961 | 0.955 | 0.958 | 0.968 | 0.953 | 0.963 | 0.962 |
| 1000 | 0.964 | 0.962 | 0.954 | 0.959 | 0.955 | 0.954 | 0.955 | 0.96 | 0.963 | 0.966 |
| 10000 | 0.968 | 0.965 | 0.965 | 0.963 | 0.974 | 0.966 | 0.956 | 0.965 | 0.965 | 0.97 |
