# Supplementary material for: rSeqDiff: Detecting Differential Isoform Expression from RNA-Seq Data Using Hierarchical Likelihood Ratio Test
Source: PLoS One. 2013 Nov 18;8(11):e79448. doi: 10.1371/journal.pone.0079448 (PMC3832546; doi:10.1371/journal.pone.0079448)
Supplement: Table S2 — Summary of true classification rate under model 1 in simulations. (DOC) [file pone.0079448.s007.doc]

**Table S2. Summary of true classification rate under model 1.**

| *τ_1_ G* | 0.01 | 0.02 | 0.05 | 0.1 | 0.2 | 0.3 | 0.4 | 0.45 | 0.49 | 0.5 |
| --- | --- | --- | --- | --- | --- | --- | --- | --- | --- | --- |
| 0.1 | 0.426 | 0.404 | 0.406 | 0.359 | 0.216 | 0.078 | 0.031 | 0.008 | 0.012 | 0.02 |
| 1 | 0.998 | 0.997 | 0.999 | 0.996 | 0.992 | 0.961 | 0.36 | 0.097 | 0.022 | 0.014 |
| 10 | 0.998 | 0.997 | 0.99 | 0.981 | 0.977 | 0.971 | 0.969 | 0.79 | 0.049 | 0.029 |
| 100 | 0.982 | 0.977 | 0.975 | 0.977 | 0.973 | 0.973 | 0.976 | 0.971 | 0.374 | 0.032 |
| 1000 | 0.973 | 0.963 | 0.97 | 0.976 | 0.973 | 0.971 | 0.975 | 0.977 | 0.965 | 0.02 |
| 10000 | 0.973 | 0.985 | 0.974 | 0.979 | 0.972 | 0.967 | 0.979 | 0.978 | 0.982 | 0.013 |
