# Supplementary material for: rSeqDiff: Detecting Differential Isoform Expression from RNA-Seq Data Using Hierarchical Likelihood Ratio Test
Source: PLoS One. 2013 Nov 18;8(11):e79448. doi: 10.1371/journal.pone.0079448 (PMC3832546; doi:10.1371/journal.pone.0079448)
Supplement: Table S3 — Summary of true classification rate under model 2 in simulations. (DOC) [file pone.0079448.s008.doc]

**Table S3. Summary of true classification rate under model 2 in simulations.**

| *Δψ* ***G*** | 0.01 | 0.05 | 0.1 | 0.2 | 0.3 | 0.4 | 0.5 | 0.6 | 0.75 | 0.8 | 0.9 | 0.95 | 0.99 |
| --- | --- | --- | --- | --- | --- | --- | --- | --- | --- | --- | --- | --- | --- |
| 0.1 | 0 | 0 | 0 | 0 | 0 | 0 | 0 | 0 | 0 | 0 | 0 | 0 | 0 |
| 1 | 0.011 | 0.011 | 0.008 | 0.022 | 0.039 | 0.035 | 0.05 | 0.081 | 0.136 | 0.144 | 0.219 | 0.233 | 0.272 |
| 10 | 0.016 | 0.033 | 0.057 | 0.138 | 0.246 | 0.386 | 0.55 | 0.692 | 0.822 | 0.883 | 0.93 | 0.949 | 0.956 |
| 100 | 0.037 | 0.114 | 0.294 | 0.665 | 0.892 | 0.936 | 0.967 | 0.975 | 0.989 | 0.987 | 0.994 | 0.993 | 0.995 |
| 1000 | 0.063 | 0.54 | 0.881 | 0.975 | 0.987 | 0.998 | 0.994 | 0.999 | 0.996 | 0.997 | 1 | 1 | 1 |
| 10000 | 0.301 | 0.952 | 0.989 | 1 | 1 | 0.999 | 1 | 1 | 1 | 0.999 | 1 | 1 | 1 |
