# Supplementary material for: rSeqDiff: Detecting Differential Isoform Expression from RNA-Seq Data Using Hierarchical Likelihood Ratio Test
Source: PLoS One. 2013 Nov 18;8(11):e79448. doi: 10.1371/journal.pone.0079448 (PMC3832546; doi:10.1371/journal.pone.0079448)
Supplement: Table S4 — Performance of rSeqDiff with varying read numbers. (DOC) [file pone.0079448.s009.doc]

**Tabl**e S4. Performance of rSeqDiff with varying read numbers

|  | Percentage of total reads | | | | | |
| --- | --- | --- | --- | --- | --- | --- |
|  | 5% | 10% | 25% | 50% | 75% | 100% |
| Reads > 5 | 13035 | 14251 | 16623 | 18524 | 19962 | 20882 |
| Model 0 | 8216 | 8597 | 8876 | 9021 | 9273 | 9319 |
| Model 1 | 3827 | 4530 | 6431 | 7934 | 8948 | 9805 |
| Model 2 | 992 | 1124 | 1316 | 1569 | 1741 | 1758 |
| Among 164 PCR tested genes | 78 | 102 | 121 | 143 | 148 | 150 |

This table shows the performance of rSeqDiff when using 5%, 10%, 25%, 50%, 75% and 100% of total reads (total reads are 136 million for the ESRP1 sample and 120 million for the EV samples). The numbers in the table are the number of genes detected Percentage of total reads: s for ing read numbers0000000000000000000000000000000000000000000000000000000000000000000000000000000using the corresponding percentage of reads.
