# Supplementary material for: rSeqDiff: Detecting Differential Isoform Expression from RNA-Seq Data Using Hierarchical Likelihood Ratio Test
Source: PLoS One. 2013 Nov 18;8(11):e79448. doi: 10.1371/journal.pone.0079448 (PMC3832546; doi:10.1371/journal.pone.0079448)
Supplement: Table S5 — Comparison of the estimated differentially used exon inclusion levels for the five RT-PCR validated genes between rSeqDiff and the exon-based method in Voineagu et al . (DOC) [file pone.0079448.s010.doc]

**Table S5. Comparison of the estimated differentially used exon inclusion levels for the five RT-PCR validated genes between rSeqDiff and the method used in Voineagu *et al***

| Gene | Isoform include the alternative exon | Isoforms skip the alternative exon | %inc ASD* (rSeqDiff/exon-based) | %inc control* (rSeqDiff/exon-based) | %inc difference* (rSeqDiff/exon-based) |
| --- | --- | --- | --- | --- | --- |
| RPN2 | NM_001135771 | NM_002951 | 23.8% / 22% | 62.4% / 69% | -38.6% / -47% |
| EHBP1 | NM_015252, NM_001142614 | NM_001142615, NM_001142616 | 62.9% / 51% | 93.8% / 90% | -30.9% / -39% |
| GRIN1 | NM_001185091, NM_001185090 | NM_021569, NM_007327, NM_000832 | 14.2% / 8% | 38.7% / 44% | -24.5% / -36% |
| SORBS1 | NM_001034954, NM_001034955 | NM_001034956 | 18.5% / 1% | 49.1% / 35% | -30.6% / -34% |
| NRCAM | NM_001193583, NM_00193582 | NM_005010, NM_001193584 | 28.8% / 33% | 62.1% / 65% | -33.3% / -32% |

*%inc: exon inclusion level
