# Supplementary material for: rSeqDiff: Detecting Differential Isoform Expression from RNA-Seq Data Using Hierarchical Likelihood Ratio Test
Source: PLoS One. 2013 Nov 18;8(11):e79448. doi: 10.1371/journal.pone.0079448 (PMC3832546; doi:10.1371/journal.pone.0079448)
Supplement: Table S6 — Comparison of differential spliced genes across biological replicates in the ASD dataset. (DOC) [file pone.0079448.s011.doc]

**Table S6. Comparison of differential spliced genes across biological replicates in the ASD dataset**

| ASD sample ID | Control sample ID | Number of DS genes identified | Number of DS genes overlapping with Pool (%) | PCC-ASD | PCC-control |
| --- | --- | --- | --- | --- | --- |
| A_AN09730_22 | C_AN00142_09 | 1614 | 1352 (83.8%) | 0.803 | 0.825 |
| A_AN17777_41 | C_AN10028_41 | 2015 | 1610 (79.9%) | 0.876 | 0.841 |
| A_AN19511_09 | C_AN12240_41 | 1212 | 989 (81.6%) | 0.812 | 0.832 |

DS: differential spliced;

Pool: separately pooling the reads of the biological replicates in ASD and control, which is the way that we handled biological replicates as described in the main text (1769 DS genes are identified);

PCC-ASD: Pearson Correlation Coefficient of the estimated abundance of the overlapping DS transcripts with Pool in ASD;

PCC-control: Pearson Correlation Coefficient of the estimated abundance of the overlapping DS transcripts with Pool in control.
