# Supplementary material for: rSeqDiff: Detecting Differential Isoform Expression from RNA-Seq Data Using Hierarchical Likelihood Ratio Test
Source: PLoS One. 2013 Nov 18;8(11):e79448. doi: 10.1371/journal.pone.0079448 (PMC3832546; doi:10.1371/journal.pone.0079448)
Supplement: Text S1 — Supplementary methods and results. (DOC) [file pone.0079448.s018.doc]

**Text S1. Supplementary methods and results.**

**Contents**

1. **Methods for estimating the MLEs and their confidence intervals for the three models**
2. **Usage of the rSeqDiff package**
3. **Results of simulation studies**
4. **Supplemental methods and results for the analysis of the ESRP1 dataset**
5. **Supplemental methods and results for the analysis of the ASD dataset**

**References**

**1. Methods for estimating the MLEs and their confidence intervals for the three models**

1.1. Estimating the MLEs

The original likelihood functions for the three models are:

Model 0:

$L_{0}\left(\tilde{\theta}_{0}|N_{1},N_{2},\ldots,N_{k}\right)=\prod_{k=1}^{K} \prod_{j=1}^{J} \frac{\left(\tilde{\theta}_{0}\cdot a_{kj}\right)^{n_{kj}}e^{-\tilde{\theta}_{0}\cdot a_{kj}}}{n_{kj}!}$ (1)

Model 1:

$L_{1}\left(\tilde{\theta}_{1},\tau|N_{1},N_{2},\ldots,N_{K}\right)=\prod_{k=1}^{K} \prod_{j=1}^{J} \frac{[(\tau_{k}\tilde{\theta}_{1})\cdot a_{kj}]^{n_{kj}}e^{-(\tau_{k}\tilde{\theta}_{1})\cdot a_{kj}}}{n_{kj}!}$ (2)

Model 2:

$L_{2}\left(\theta|N_{1},N_{2},\ldots,N_{K}\right)=\prod_{k=1}^{K} \prod_{j=1}^{J} \frac{\left(\theta_{k}\cdot a_{kj}\right)^{n_{kj}}e^{-\theta_{k}\cdot a_{kj}}}{n_{kj}!}$ (3)

We adopt the two data reduction techniques introduced in : (i) We take only read types with non-zero mapped reads and further group them to form larger read categories; (ii) For each condition *k*, we compute the total sampling rate for each isoform *i* $w_{ki}\mathop{=}\limits^{def} \sum_{j=1}^{J} a_{kij}$ (denote $W_{k}=\left[w_{k1},\text{ }w_{k2},\ldots w_{kJ}\right]^{T}$as the total sampling rate vector for all isoforms) without enumerating each particular sampling rate $a_{kij}$. The reduced likelihood functions and the EM algorithm that calculate the MLE for each model are derived below.

*1.1.1 Model 0*

The likelihood for model 0 [equation (2)] can be reduced to

$\begin{matrix} L_{0}\left(\tilde{\theta}_{0}|N_{1},N_{2},\ldots,N_{k}\right)=\prod_{k=1}^{K} \left[\prod_{n_{kj}>0} \frac{\left(\tilde{\theta}_{0}\cdot a_{kj}\right)^{n_{kj}}}{n_{kj}!}\prod_{n_{kj}=0} \frac{\left(\tilde{\theta}_{0}\cdot a_{kj}\right)^{n_{kj}}}{n_{kj}!}\prod_{j=1}^{J} e^{-\tilde{\theta}_{0}\cdot a_{kj}}\right] \\ \\ =\prod_{k=1}^{K} \left[\prod_{n_{kj}>0} \frac{\left(\tilde{\theta}_{0}\cdot a_{kj}\right)^{n_{kj}}}{n_{kj}!}\prod_{j=1}^{J} e^{-\tilde{\theta}_{0}\cdot a_{kj}}\right] \\ \\ =\prod_{k=1}^{K} \left[\prod_{n_{kj}>0} \frac{\left(\tilde{\theta}_{0}\cdot a_{kj}\right)^{n_{kj}}}{n_{kj}!}\prod_{j=1}^{J} e^{-\sum_{i=1}^{I} \tilde{\theta}_{0i}a_{kij}}\right] \\ \\ =\prod_{k=1}^{K} \left[\prod_{n_{kj}>0} \frac{\left(\tilde{\theta}_{0}\cdot a_{kj}\right)^{n_{kj}}}{n_{kj}!}\prod_{I=1}^{I} e^{-\tilde{\theta}_{0i}\sum_{j=1}^{J} a_{kij}}\right] \\ \\ =\prod_{k=1}^{K} \left[\prod_{n_{kj}>0} \frac{\left(\tilde{\theta}_{0}\cdot a_{kj}\right)^{n_{kj}}}{n_{kj}!}\prod_{i=1}^{I} e^{-\tilde{\theta}_{0i}w_{ki}}\right] \end{matrix}$ (4)

The MLE of $\tilde{\theta}_{0}$ can be obtained by maximizing the log-likelihood of (4), which in the matrix form is:

$\widehat{\tilde{\theta}}_{0,MLE}=\mathop{\mathrm{argmax}}\limits_{\Theta} \mathop{sum}\limits_{K} [N_{k}^{T}\log\left(A_{k}^{T}\tilde{\theta}_{0}\right)-W_{k}^{T}\tilde{\theta}_{0}]$ (5)

subjects to the constraint $\tilde{\theta}_{0}>0$ .

We define *n_kij_* as the number of reads mapped to type *s_j_* from isoform *i* in condition *k*, regard it as hidden data, and derive the EM algorithm solving (5) as follows

E-step: $n_{kij}^{(m+1)}=\frac{n_{kj}\tilde{\theta}_{0i}^{(m)}a_{kij}}{\sum_{i=1}^{I} \tilde{\theta}_{0i}^{(m)}a_{kij}}$,

M-step: $\tilde{\theta}_{0i}^{(m+1)}=\frac{\sum_{k=1}^{K} \sum_{j=1}^{J} n_{kij}^{(m+1)}}{\sum_{k=1}^{K} \sum_{j=1}^{J} a_{kij}}$,

where $\tilde{\theta}_{0i}$ is the *i*th element of $\tilde{\theta}_{0}$ and the superscripts (*m*) and (*m*+1) denote the *m*th and (*m*+1)th iterations. The algorithm iterates until $\sum_{i=1}^{I} \left|\tilde{\theta}_{0i}^{(m+1)}-\tilde{\theta}_{0i}^{(m)}\right|\leq10^{-6}$.

*1.1.2 Model 1*

The likelihood for model 1 [equation (2)] can be reduced to:

$\begin{matrix} L_{1}\left(\tilde{\theta}_{1},\tau|N_{1}, N_{2},\ldots,N_{K}\right)=\prod_{k=1}^{K} \left[\prod_{n_{kj}>0} \frac{\left\{\left(\tau_{k}\tilde{\theta}_{1}\right)\cdot a_{kj}\right\}^{n_{kj}}}{n_{kj}!}\prod_{n_{kj}=0} \frac{\left\{\left(\tau_{k}\tilde{\theta}_{1}\right)\cdot a_{kj}\right\}^{n_{kj}}}{n_{kj}!}\prod_{j=1}^{J} e^{-\left(\tau_{k}\tilde{\theta}_{1}\right)\cdot a_{kj}}\right] \\ \\ =\prod_{k=1}^{K} \left[\prod_{n_{kj}>0} \frac{\left\{\left(\tau_{k}\tilde{\theta}_{1}\right)\cdot a_{kj}\right\}^{n_{kj}}}{n_{kj}!}\prod_{j=1}^{J} e^{-\left(\tau_{k}\tilde{\theta}_{1}\right)\cdot a_{kj}}\right] \\ \\ =\prod_{k=1}^{K} \left[\prod_{n_{kj}>0} \frac{\left\{\left(\tau_{k}\tilde{\theta}_{1}\right)\cdot a_{kj}\right\}^{n_{kj}}}{n_{kj}!}\prod_{j=1}^{J} e^{-\sum_{i=1}^{I} (\tau_{k}\tilde{\theta}_{1i})a_{kij}}\right] \\ \\ =\prod_{k=1}^{K} \left[\prod_{n_{kj}>0} \frac{\left\{\left(\tau_{k}\tilde{\theta}_{1}\right)\cdot a_{kj}\right\}^{n_{kj}}}{n_{kj}!}\prod_{i=1}^{I} e^{-(\tau_{k}\tilde{\theta}_{1i})\sum_{j=1}^{I} a_{kij}}\right] \\ \\ =\prod_{k=1}^{K} \left[\prod_{n_{kj}>0} \frac{\left\{\left(\tau_{k}\tilde{\theta}_{1}\right)\cdot a_{kj}\right\}^{n_{kj}}}{n_{kj}!}\prod_{i=1}^{I} e^{-\tau_{k}\tilde{\theta}_{1i}w_{ki}}\right] \end{matrix}$ (6)

The MLE of $\tilde{\theta}_{1}$ and $\tau$ can be obtained by maximizing the log-likelihood of (6), which in the matrix form is:

$\widehat{\left(\tilde{\theta}_{1},\tau\right)}_{MLE}=\mathop{\mathrm{argmax}}\limits_{\Theta,T} \mathop{sum}\limits_{K} [N_{k}^{T}\log\left(A_{k}^{T}\tilde{\theta}_{1}\right)+\left(\log\tau_{k}\right)sum\left(N_{k}^{T}\right)-\tau_{k}W_{k}^{T}\tilde{\theta}_{1}]$ (7)

subjects to constraint $\tilde{\theta}_{1}>0$ and $sum\left(\tau\right)=\sum_{k=1}^{K} \tau_{k}=1$.

Similar to model 0, we define *n_kij_* as the number of reads mapped to type *s_j_* from isoform *i* in condition *k*, and use it as the hidden data, and derive the EM algorithm solving (7) as follows:

E-step: $n_{kij}^{(m+1)}=\frac{n_{kj}\tilde{\theta}_{1i}^{(m)}a_{kij}}{\sum_{i=1}^{I} \tilde{\theta}_{1i}^{(m)}a_{kij}}$,

M-step: $\tau_{k}^{(m+1)}=\frac{\sum_{j=1}^{J} n_{kj}}{\sum_{i=1}^{I} \sum_{i=1}^{I} \tilde{\theta}_{1i}^{(m)}a_{kij}}$,

$\tilde{\theta}_{1i}^{(m+1)}=\frac{\sum_{k=1}^{K} \sum_{j=1}^{J} n_{kij}^{(m+1)}}{\sum_{k=1}^{K} \sum_{j=1}^{J} a_{kij}\tau_{k}^{(m+1)}}$,

where $\tilde{\theta}_{1i}$ is the *i*th element of $\tilde{\theta}_{i}$**,** *τ_k_* is the *k*th element of *τ*; and the superscripts (*m*) and (*m*+1) denote the *m*th and (*m*+1)th iterations. The algorithm iterates until $\sum_{\text{i}=1}^{\text{I}} \left|\tilde{\text{θ}}_{0\text{i}}^{(\text{m}+1)}-\tilde{\text{θ}}_{0\text{i}}^{(\text{m})}\right|\leq10^{-6}$ and $\sum_{\text{k}=1}^{\text{K}} \left|\text{τ}_{\text{k}}^{(\text{m}+1)}-\text{τ}_{\text{k}}^{(\text{m})}\right|\leq10^{-6}$ (note the linear constraint $\sum_{k=1}^{K} \tau_{k}=1$ is automatically satisfied in the EM algorithm).

*1.1.3 Model 2*

The likelihood for model 2 [equation (3)] can be reduced to:

$\begin{matrix} L_{2}\left(\text{θ}|N_{1},N_{2},\ldots,N_{k}\right)=\prod_{k=1}^{K} \left[\prod_{n_{kj}>0} \frac{\left(\text{θ}_{k}\cdot a_{kj}\right)^{n_{kj}}}{n_{kj}!}\prod_{n_{kj}=0} \frac{\left(\text{θ}_{k}\cdot a_{kj}\right)^{n_{kj}}}{n_{kj}!}\prod_{j=1}^{J} e^{-\text{θ}_{k}\cdot a_{kj}}\right] \\ \\ =\prod_{k=1}^{K} \left[\prod_{n_{kj}>0} \frac{\left(\text{θ}_{k}\cdot a_{kj}\right)^{n_{kj}}}{n_{kj}!}\prod_{j=1}^{J} e^{-\text{θ}_{k}\cdot a_{kj}}\right] \\ \\ =\prod_{k=1}^{K} \left[\prod_{n_{kj}>0} \frac{\left(\text{θ}_{k}\cdot a_{kj}\right)^{n_{kj}}}{n_{kj}!}\prod_{j=1}^{J} e^{-\sum_{i=1}^{I} \text{θ}_{ki}a_{kij}}\right] \\ \\ =\prod_{k=1}^{K} \left[\prod_{n_{kj}>0} \frac{\left(\text{θ}_{k}\cdot a_{kj}\right)^{n_{kj}}}{n_{kj}!}\prod_{I=1}^{I} e^{-\text{θ}_{ki}\sum_{j=1}^{J} a_{kij}}\right] \\ \\ =\prod_{k=1}^{K} \left[\prod_{n_{kj}>0} \frac{\left(\text{θ}_{k}\cdot a_{kj}\right)^{n_{kj}}}{n_{kj}!}\prod_{i=1}^{I} e^{-\text{θ}_{ki}w_{ki}}\right] \end{matrix}$ (8)

To obtain the MLE of *θ*, note that all the $\theta_{k}$’s are independent and the likelihood function (8) can be factorized as the product of the likelihood function for each condition *k*. Therefore, we can compute the MLE of each $\theta_{k}$ in condition *k* as

$\hat{\theta}_{k,MLE}=\mathop{\mathrm{argmax}}\limits_{\Theta_{k}} N_{k}^{T}\log\left(A_{k}^{T}\theta_{k}\right)-W_{k}^{T}\theta_{k}$ (9)

subjects to constraint $\theta_{k}>0$.

To solve (9), similar to model 0, we define *n_kij_* as the number of reads mapped to type *s_j_* from isoform *i* in condition *k*, and use it as the hidden data, and derive the EM algorithm solving (7) as follows

E-step: $n_{kij}^{(m+1)}=\frac{n_{kj}\theta_{ki}^{(m)}a_{kij}}{\sum_{i=1}^{I} \theta_{ki}^{(m)}a_{kij}}$,

M-step: $\theta_{ki}^{(m+1)}=\frac{\sum_{k=1}^{K} \sum_{j=1}^{J} n_{kij}^{(m+1)}}{\sum_{k=1}^{K} \sum_{j=1}^{J} a_{kij}}$,

where $\theta_{ki}$ is the *i*th element of $\theta_{k}$ and the superscripts (*m*) and (*m*+1) denote the *m*th and (*m*+1)th iterations. The algorithm iterates until $\sum_{i=1}^{I} \left|\theta_{ki}^{(m+1)}-\theta_{ki}^{(m)}\right|\leq10^{-6}$.

Then the MLE of *θ* is given by:


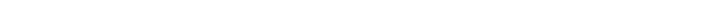


1.2. Estimating the confidence intervals for the MLEs

To estimate the confidence intervals for the MLEs, we apply similar strategy of importance sampling as introduced in . We approximate the distribution of the MLEs by multivariate *t* distributions of $df=5$ with mean as the point estimations of the MLEs and covariance matrix as the inverse of observed Fisher information matrix. We generate 50,000 random samples $\Theta^{(1)},\text{ }\Theta^{(2)},\ldots,\text{ }\Theta^{(50000)}$ from this density and associate with each sample an importance weight:

$w^{\left(i\right)}=\frac{L\left(\text{Θ}^{\left(i\right)}\right)}{q\left(\text{Θ}^{\left(i\right)}\right)}$,

where $L\left(\Theta^{\left(i\right)}\right)$ is the likelihood at $\text{Θ}^{\left(i\right)}$ and $q\left(\text{Θ}^{\left(i\right)}\right)$ is the density of the above multivariate *t* distribution at $\text{Θ}^{\left(i\right)}$.

Using these weighted samples, we can estimate the posterior probability of any event A as:


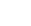

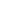


The 95% confidence interval of the MLE is computed as an interval around the point estimation of the MLE that contains 95% of the posterior weight.

**2. Usage of the rSeqDiff package**

In this section, we briefly outline the analysis pipeline of the rSeqDiff package, which is used in the analyses of the ESRP and ASD RNA-Seq datasets. Detailed instructions for the usage of the package are provided on the website of the package. Since rSeqDiff is an isoform-based method, it can infer complex multiple splicing of isoforms, including exon skipping, alternative splicing of 5’ and/or 3’ sites, alternative splicing of mutually exclusive exons and alternative splicing of retained introns.

Figure S1 illustrates the pipeline of rSeqDiff, which includes the following three steps starting from raw RNA-Seq read data in FASTQ or FASTA format: (1) For each condition, map the reads to the transcript sequences. We use Bowtie for read mapping in our analyses of the ESRP and ASD RNA-Seq datasets. After this step, the sequence alignment files in either SAM format or Eland-multiple format should be generated. (2) Use rSeq (a software tool for RNA-Seq data analysis developed by the authors based on the methods described in . Detailed description of its usage is provided on its website ) to process the sequence alignment files to generate the “.sampling_rates” files which contain the sampling rate matrix *A_k_*, the read count vector *N_k_* and the sum of sampling-rate vector *W_k_* for each gene. (rSeq can also generate BED format files that can be used for visualizing the reads mapped to a particular gene in USCS genome browser or CisGenome Browser . See for details). (3) Process the “.sampling_rates” files using rSeqDiff and obtaining the list of genes classified to each of the three models and the estimates of gene and isoform abundances for each gene.

**3. Results of simulation studies**

We study the type I error and statistical power of the proposed hLRT through simulations. For each of the three models, we simulate the isoform abundance of a gene under that model and calculate the proportions of the simulated samples that have been correctly classified to the true underlying model (i.e., true classification rate). Default significance level α=0.05 is used throughout the simulations.

Figure S2 shows a hypothetical gene that is used in the simulations. The gene has three exons and two isoforms, with the middle exon of length 60 bp being differentially used. We assume two hypothetical conditions being compared with 50 million reads and 55 million reads, respectively. We use $\left(\theta_{11},\theta_{12}\right)^{T}$ and $\left(\theta_{21},\theta_{22}\right)^{T}$to denote the isoform abundances under the two conditions. The sampling rate matrices for the two conditions are:

$A_{1}=\left[\begin{matrix} 1200 & 60 & 600 \\ 1200 & 0 & 600 \end{matrix}\right]\times50\times10^{6}/10^{9}=\left[\begin{matrix} 60 & 3 & 30 \\ 60 & 0 & 30 \end{matrix}\right]$,

$A_{2}=\left[\begin{matrix} 1200 & 60 & 600 \\ 1200 & 0 & 600 \end{matrix}\right]\times55\times10^{6}/10^{9}=\left[\begin{matrix} 66 & 3.3 & 66 \\ 66 & 0 & 66 \end{matrix}\right]$.

*Simulations under model 0.* Let *G* denote the gene abundance and *ψ* denote the abundance ratio between isoform 1 and *G*. The abundances of the two isoforms under the two conditions are given by: $\theta_{11}=\theta_{21}=G\psi$ and $\theta_{12}=\theta_{22}=G\left(1-\psi\right)$. *G* and *ψ* are the factors varying in the simulation, with *G*=0.1, 1, 10, 100, 1000, 10000 and *ψ*=0.01, 0.02, 0.05, 0.1, 0.2, 0.3, 0.4, 0.45, 0.49, 0.5. For each combination of *G* and *ψ*, we calculate *θ* and simulate 1000 random samples of read counts according to $n_{kij}\text{～}Poisson\left(a_{kij}\theta_{ki}\right)$ with $k=1,2;\text{ }i=1,2;\text{ }j=1,2,3$.

We run the algorithm with the 1000 samples as the input and calculate the rates that the samples are correctly classified as model 0. Table S1 shows the true classification rate. This rate is above 95% for all cells, which shows that the type I error rate is controlled at 0.05.

*Simulations under model 1.* Let *G* denote the total gene abundance of the two conditions, $\tau_{1}$ and $\tau_{2}$ denote the ratios of the isoform abundance between the two conditions with $\tau_{1}+\tau_{2}=1$; $\psi$ denote abundance ratio between isoform 1 and the gene fixing the condition. Then the abundance of the two isoforms under the two conditions is given by:

$\theta_{11}=G\tau_{1}\psi,\text{ }\theta_{12}=G\tau_{1}\left(1-\psi\right)$ (1.1)

$\theta_{21}=G\tau_{2}\psi,\text{ }\theta_{22}=G\tau_{2}\left(1-\psi\right)$ (1.2)

*G* and $\tau_{1}$ are the parameters varying in the simulations, with *G*=0.1, 1, 10, 100, 1000, 10000 and $\tau_{1}$=0.01, 0.02, 0.05, 0.1, 0.2, 0.3, 0.4, 0.45, 0.49, 0.5 (When $\tau_{1}$=0.5, the true underlying model degenerates to model 0). For each combination of *G* and $\tau_{1}$, 1000 random samples of read counts were simulated according to the following steps:

1. simulate $\psi\text{～}uniform\left(0,\text{ }0.5\right)$;
2. calculate $\theta_{11}$, $\theta_{12}$, $\theta_{21}$ and $\theta_{22}$ based on equations (1.1) and (1.2);
3. simulate $n_{kij}\text{～}Poisson\left(a_{kij}\theta_{ki}\right)$, with $k=1,2;\text{ }i=1,2;\text{ }j=1,2,3$.

We repeat the above steps 1000 times and obtain 1000 random samples of read counts from the true underlying model 1 for each combination of *G* and $\tau_{1}$. The observed true classification rate is summarized in Table S2. As expected, the true classification rate depends on *G* and $\tau_{1}$: For genes with relative low abundance ($0.1\leq G\leq10$),when *G* is fixed and $\tau_{1}$ increases close to 0.5, the true underlying model tends to degenerate to model 0 and the power decreases consequently; For genes with relative high abundance ($G\geq100$), the proposed hLRT has a high power to identify genes from the true underlying model 1 across a wide range of $\tau_{1}$. Overall, the power is reasonably high for $G\geq1$.

*Simulations under model 2.* Let *G* denote the total gene abundance of the two conditions (i.e. *G* is the sum of all $\theta$s); $\tau_{1}$ and $\tau_{2}$ denote the ratios of the gene abundance between the two conditions with $\tau_{1}+\tau_{2}=1$; $\psi_{1}$ and $\psi_{2}$ denote abundance ratio between isoform 1 and the gene in condition 1 and condition 2, respectively; $\Delta\psi$ denote the difference between $\psi_{1}$ and $\psi_{2}$ (i.e. $\Delta\psi=\psi_{1}-\psi_{2}$. Note when $\Delta\psi=0$, the true underlying model degenerates to model 1; when $\Delta\psi=0$ and $\tau_{1}=\tau_{2}=0.5$, the true underlying model degenerates to model 0). Then the abundance of the two isoforms under the two conditions is given by:

$\theta_{11}=G\tau_{1}\psi_{1},\text{ }\theta_{12}=G\tau_{1}\left(1-\psi_{1}\right)$ (2.1)

$\theta_{21}=G\tau_{2}\psi_{2},\text{ }\theta_{22}=G\tau_{2}\left(1-\psi_{2}\right)$ (2.2)

*G* and $\Delta\psi$ are the factors varying in the simulation, with *G*=0.1, 1, 10, 100, 1000, 10000 and $\Delta\psi$=0.01, 0.05, 0.1, 0.2, 0.3, 0.4, 0.5, 0.6, 0.75, 0.8, 0.9, 0.95, 0.99. For each combination of *G* and$\Delta\psi$, 1000 random samples of read counts were simulated according to the following steps:

1. simulate $\psi_{1}\text{～}uniform\left(\Delta\psi,\text{ }1\right)$;
2. calculate $\psi_{2}$: $\psi_{2}=\psi_{1}-\Delta\psi$;
3. simulate $\tau_{1}\text{～}uniform\left(0,\text{ }0.5\right)$ ;
4. calculate $\tau_{2}$: $\tau_{2}=1-\tau_{1}$;
5. calculate $\theta_{11}$, $\theta_{12}$, $\theta_{21}$and $\theta_{22}$ based on equations (2.1) and (2.2);
6. simulate $n_{kij}\text{～}Poisson\left(a_{kij}\theta_{ki}\right)$, with $k=1,2;\text{ }i=1,2;\text{ }j=1,2,3$.

We repeated the above steps 1000 times and obtained 1000 random samples of read counts from the true underlying model 2 for each combination of *G* and $\Delta\psi$. The true classification rate is summarized in Table S3. As expected, when *G* is fixed and $\tau_{1}$ decreases close to 0, the true underlying model tends to degenerate to model 1. Therefore the power decreases consequently. Overall, the power is reasonably high for $G\geq1$.

In summary, the simulations show that the proposed hLRT approach has well controlled type I error at α=0.05 and decent statistical power for detecting differential expression and differential splicing for genes with moderate to high abundance. In particular, the hLRT still has good power in the situation where the gene is lowly expressed in one condition but moderately or highly expressed in the other conditions. In that case, the gene will be classified as model 1 (differential expression but no differential splicing - see simulations under model 1). When $\tau_{1}$ is small, it represents the situation that the gene and its isoforms are lowly expressed in condition 1 but highly or lowly expressed in condition 2. The lack of power to detect differential expression or splicing occurs when the gene is expressed lowly in both conditions. This is well expected and is an inherent problem in the differential analysis from RNA-Seq data. In real data analysis, genes with very low expression levels in all the conditions are usually filtered out prior to the analysis. By default, rSeqDiff filters out genes with less than 5 reads in all the conditions.

**4. Supplemental methods and results for the analysis of the ESRP1 dataset**

This dataset was published in Shen *et al* , where 76 bp single end RNA-Seq experiments were performed on the MDA-MB-231 cell line with ectopic expression of the ESRP1 gene and an empty vector (EV) as control. The resulting dataset contains 136 million reads for the ESRP1 sample and 120 million reads for the EV sample from Illumina GA II sequencer, and the dataset is available at the NCBI SRA depository (accession numbers SRR436885 and SRR436886). For the 164 RT-PCR tested alternative exons, their genome coordinate, junctions read counts, *ψ_ESRP1_*, *ψ_EV_* and *Δψ* values from MATS and RT-PCR are all provided in . As Shen *et al* mentioned in , the first 50bp segments of the reads had a high mapping rate to the human genome and the last 25bp segment had a much lower mapping rate, so they used the first 50bp of each read for their analysis. To make our analysis comparable with theirs, we also use the first 50bp of each read in our analysis.

For the analysis using rSeqDiff with its default settings, referred as rSeqDiff (all reads) in the main text, we first map the reads to Ensembl transcript annotations of the human genome (hg19, see ) using Bowtie with up to 3bp mismatches . Then we use rSeq to generate the “.sampling_rates” files that contain the sampling rate matrix *A_k_*, the read count vector *N_k_* and the sum of sampling-rate vector *W_k_* of each gene for both the two conditions (Figure S1). Next we apply rSeqDiff to the “.sampling_rates” files with its default settings with significance level α=0.05 for the hLRT and genes with less than 5 reads in both conditions are filtered out. For the analysis of Cuffdiff 2, we exactly follow the pipeline described in the user manual . Briefly, we first map the reads to Ensembl transcript annotations of the human genome, and then run Cuffdiff 2 with its default settings . The isoform structure of the genes and the reads mapped to the genes are visualized using CisGenome Browser .

Below are some supplementary results for the analysis of the ESRP1 dataset: Table S4 shows performance of rSeqDiff with varying read numbers, where the numbers in the table are the number of genes detected by rSeqDiff using the corresponding percentage of reads; Figure S3 is the scatter plot corresponding to Table S4. We can see (i) the number of significant differential expressed or spliced genes increases with the number of total reads (sequencing depth), which is expected. (ii) The number of significant events goes up with sequencing depth in a plateau pattern. In general, using 30 million of reads (about 25% of total reads) can recover about 80% of the significant events detected by using total reads.

**5. Supplemental methods and results for the analysis of the ASD dataset**

This dataset was published in Voineagu *et al* , where 73bp single-end RNA-Seq experiments were performed on three autistic brain samples with down-regulated A2BP1 gene levels (a.k.a. FOX1, an important neuronal specific splicing factor that regulates alternative splicing in the brain) and 3 control brain samples with normal A2BP1 levels using Illumina GA II sequencer. The data is available at the NCBI SRA depository (accession number SRP007483) . The authors of used the Basic Local Alignment Tool (BLAT) to align the reads to their cDNA-derived alternative splicing junctions database and applied Fisher’s exact test and Bonfeeroni-Hochberg correction to identify differentially spliced exons associated with autism, which is an exon-based method similar to MATS. As the authors mentioned, they separately pooled the reads for ASD and control to generate sufficient read coverage for the quantitative analysis of alternative splicing events. To make the results comparable, we also separately pooled the reads for ASD and control in our analysis.

For the analysis with rSeqDiff, we first map the reads to UCSC transcript annotations of the human genome (hg19) using Bowtie with up to 3bp mismatches . Then we use rSeq to generate the “.sampling_rates” files that contain the sampling rate matrix *A_k_*, the read count vector *N_k_* and the sum of sampling-rate vector *W_k_* of each gene for both the two conditions (Figure S1). Next we apply rSeqDiff to the “.sampling_rates” files with its default settings, where significance level α=0.05 for the hLRT and genes with less than 5 reads in both conditions are filtered out. For the analysis with Cuffdiff 2, we exactly follow the pipeline described in its user manual . Briefly, we first map the reads to UCSC transcript annotations of the human genome, and then run Cuffdiff 2 with its default settings . The isoform structure of the genes and the reads mapped to the genes are visualized using CisGenome Browser .

The tests of the enrichment of gene ontology (GO) terms, functional categories, genetic association disease classes and the tissue expression pattern of the differentially spliced genes are performed using the DAVID database (http://david.abcc.ncifcrf.gov/) .

Below are some supplementary results for the analysis of the ASD dataset: Figure S4 shows two scatter plots for examining differential expression and differential splicing, respectively. Figure S4-A shows the scatter plot of the p values from the likelihood ratio test between model 1 and 0 v.s. the log2 fold changes of the estimated gene abundance (“volcano plot”), which can be used for visualizing differential expression of each gene; Figure S4-B shows the scatter plot of the p values from the likelihood ratio test between model 2 and 0 v.s. the T values for all genes, which can be used for visualizing differential splicing of each gene; Figure S5 shows the scatter plot of the log 2 fold changes of transcript abundances between ASD and control samples estimated by rSeqDiff and Cuffdiff 2 (genes with low read counts or failed to be tested by Cuffdiff 2 are excluded); Table S5 compares the estimated differentially used exon inclusion levels for the five RT-PCR validated genes between rSeqDiff and the exon-based method in ; Table S6 is the comparison of differential spliced genes across biological replicates.

**References**
